# Australasian Podiatry Conference 2017: Melbourne, Australia. 24–26 May 2017

**DOI:** 10.1186/s13047-017-0212-7

**Published:** 2017-08-03

## Oral Presentation

### O1 Lower leg and foot contributions to turnout in university-level female ballet dancers: a preliminary investigation

#### Sarah Carter^1,2^, Alan Bryant^1^, Luke Hopper^2^

##### ^1^Podiatric Medicine Unit, School Of Surgery, The University Of Western Australia, Crawley, Western Australia, Australia; ^2^Western Australian Academy Of Performing Arts, Edith Cowan University, Mt Lawley, Western Australia, Australia

###### **Correspondence:** Sarah Carter

**Objectives**

Hip external rotation measures in functional turnout has received extensive examination in dance science. However, the relationship between the ‘below the hip’ assessments with functional turnout is poorly understood. We hypothesis that dancers with greater passive external tibiofemoral rotation (pTFR) and/or pronated stance will have a greater functional turnout angle.

**Method**

Nineteen female university-level classical ballet and modern dance students (mean age 17.9 ± 0.9 years) volunteered in this descriptive correlational study. All dancers were injury free and signed a consent form prior to data collection. Navicular drop, Foot Posture Index (FPI), pTFR and functional turnout were measured for the participants’ right and left lower limbs. Measures of pronation were conducted with the dancer standing parallel and turnout. All clinical measures demonstrated excellent reliability, ICC 0.90–0.93. A multiple linear regression model was used to estimate the amount of variance in functional turnout which can be explained by the measured variables.

**Results**

The stepwise multiple linear regression model analyses revealed a weak relationship between pTFR and functional turnout, with the latter accounting for approximately 19.0% variance of functional turnout. Spearman’s rho correlation analysis revealed a moderate negative relationship between pTFR and FPI in functional turnout (ρ = −0.47, P = 0.043). Suggesting dancers with limited tibiofemoral rotation recruited pronation about the foot/ankle complex to further increase their functional turnout angle.

**Conclusions**

Our findings suggest dancers used variable amounts of motion at the anatomical locations depending on their functional and anatomical capability. Ongoing research would benefit from in situ measures of dancers’ lower leg contributions to functional turnout such as that provided by modern three- dimensional biomechanical evaluations.

### O2 Kinematic repeatability of a multi-segment foot model for dance

#### Sarah Carter^1,2^, Nahoko Sato^3^, Luke Hopper^2^

##### ^1^Podiatric Medicine Unit, School Of Surgery, The University Of Western Australia, Crawley, Western Australia, Australia; ^2^Western Australian Academy Of Performing Arts, Edith Cowan University, Mt Lawley, Western Australia, Australia, ^3^Nagoya Gakuin University, Seto, Aichi, Japan

###### **Correspondence:** Sarah Carter

**Objectives**

The purpose of this study was to determine the intra and inter-assessor repeatability of a modified Rizzoli Foot Model marker set-up by analysing ballet dancers during flex-pointé-flex movements and static ballet positions.

**Method**

Six university-level ballet dancers performed the movements; parallel stance, turnout plié, turnout stance, turnout rise and flex-pointé-flex. A twelve-camera motion capture system were used to track fourteen reflective markers and one triad on the following segments: shank, entire foot, hindfoot, midfoot, forefoot and hallux. A repeated-measure design was used with each participant undergoing four data collection sessions; i.e. two sessions were conducted by each researcher over two consecutive days. Variability of the 3D segment rotations and planar angles were determined using intra-class correlation coefficients (ICC) for the intra and inter-assessor repeatability.

**Results**

Intra and inter-assessor reliability demonstrated excellent (ICC ≥ 0.75) repeatability for the 1^st^ metatarsophalangeal joint in the sagittal plane. Intra-assessor reliability demonstrated excellent (ICC ≥ 0.75) repeatability during flex-pointé-flex across all inter-segmental angles except for the tibia- hindfoot and hindfoot-midfoot frontal planes. Inter-assessor repeatability ranged from poor to excellent (0.5 > ICC ≥ 0.75) for the 3D segment rotations. The most repeatable measure was the tibia-foot dorsiflexion/plantar flexion articulation whereas the least repeatable measure was the hindfoot- midfoot adduction/abduction articulation. The variation found in the inter-assessor results is likely due to inconsistencies in marker placement.

**Conclusions**

This 3D dance specific multi-segment foot model provides insight into which kinematic measures can be reliably used to ascertain in vivo technical errors and/or biomechanical abnormalities in a dancer’s foot motion.

### O3 The relationship of fear of falling with falls in adults with rheumatoid arthritis: a prospective longitudinal study

#### Angela Brenton-Rule^1^, Nicola Dalbeth^2^, Hylton B. Menz^3^, Keith Rome^1^

##### ^1^AUT University, Auckland, New Zealand; ^2^University of Auckland, Auckland, New Zealand; ^3^La Trobe University, Melbourne, Australia

###### **Correspondence:** Angela Brenton-Rule

**Objectives**

People with rheumatoid arthritis (RA) have an increased risk of falling. A fear of falling can result from falls and predicts risk of future falls in healthy older adults. The aim of this study was to evaluate the temporal relationship of fear of falling with falls, in adults with RA.

**Method**

Adults with RA were recruited from rheumatology outpatient clinics. RA characteristics, fear of falling and 12-month fall history were assessed at baseline. Fear of falling was determined using the short- form Falls Efficacy Scale-International (FES-I). Participants were then followed for 12 months, to prospectively record the occurrence of falls. Fear of falling was reassessed at the end of 12 months. Mann Whitney U tests were used to, 1) compare baseline fallers and non-fallers on FES-I score at baseline and 12 months, 2) compare fallers and non-fallers over the 12-month follow-up period on FES-I score at baseline and 12 months.

**Results**

201 participants completed the baseline assessment and 196 completed the 12-month follow-up. Fear of falling was reported by 81% at baseline; mean(SD) FES-I score 12(5), and 81% at 12 months; mean(SD) FES-I score 12(4). At baseline, 59% reported a 12-month fall history and 42% fell at least once during the 12-month follow-up period. Compared to non-fallers, participants with a fall history at baseline had significantly higher FES-I score at baseline (*p* = 0.002) and 12 months (*p* = 0.024). However, there was no significant difference between fallers and non-fallers over the 12-month follow- up period on FES-I score at baseline (*p* = 0.14) and 12 months (*p* = 0.21).

**Conclusions**

Fear of falling is significantly higher in people with RA who have previously fallen. However, fear of falling does not predict future falls in people with RA. The findings suggest that predictors of falls in adults with RA may differ to the general older adult population.

### O4 Exploring musculoskeletal injuries in the podiatry profession: an international cross sectional study

#### Cylie Williams^1,2^, Stefania Penkala^3^, Peter Smith^4,5^, Terry Haines^1,6^, Kelly-Ann Bowles^1^

##### ^1^Monash University, Frankston, VIC, Australia; ^2^Peninsula Health, Frankston, VIC, Australia; ^3^Western Sydney University, Penrith, NSW, Australia; ^4^Monash University, Prahran, VIC, Australia; ^5^University of Toronto, Toronto, Canada; ^6^Monash Health, Cheltenham, VIC, Australia

###### **Correspondence:** Cylie Williams

This abstract is not included here as it has already been published.

### O5 Health related quality of life of children with calcaneal apophysitis: child and parent perceptions

#### Alicia James^1,2^, Cylie Williams^1,2^, Terry Haines^2,3^

##### ^1^Peninsula Health, Frankston, Victoria, Australia; ^2^Monash University, Peninsula Campus, Frankston, Victoria, Australia; ^3^Monash Health, Cheltenham, Victoria, Australia

###### **Correspondence:** Alicia James

This abstract is not included here as it has already been published.

### O6 Does a non-weight bearing foot position replicate the neutral calcaneal stance position in an adult population?

#### Hayley Walker, Rolf Scharfbillig, Sara Jones

##### University of South Australia, Adelaide, SA, Australia

###### **Correspondence:** Hayley Walker

**Objectives**

The Neutral Calcaneal Stance Position (NCSP), despite its known issues, is currently used as an ‘ideal’ measure, in comparison to resting. The non-weightbearing (NWB) foot as used in the Foot Mobility Magnitude may provide an alternative comparative position, if a significant correlation between NCSP and NWB positions exists.

**Method**

Eighty participants were recruited from the University of South Australia, Division of Health Science. Measures of total foot length (TFL) were obtained and 50 percent of TFL was marked on the dorsum of the left foot. Measures of Dorsal Arch Height (DAH) and Midfoot Width (MFW) were obtained at the 50 percent TFL mark. Measures were obtained by two examiners in a NCSP and NWB position using apparatus as described by McPoil et al. 2009.

**Results**

Reliability analysis with Intraclass Correlation Coefficients (ICC) indicated intra-rater results of 0.90 to 0.99 (ICC) for DAH and 0.96–0.99 (ICC) for MFW and inter-rater results of 0.90 (ICC) and 0.96 (ICC) for DAH and MFW, respectively in a NWB position. Using a Pearson product-moment correlation coefficient analysis, results indicated there was a significant correlation between NCSP and NWB positions for both DAH (r = 0.82) and MFW (r = 0.86).

**Conclusions**

In conclusion, a significant correlation between the NCSP and NWB positions was evident when the measures of DAH and MFW were conducted. Therefore, clinically the NWB position can potentially replace the NCSP as the ‘ideal’ position in comparison to resting by using DAH and MFW, when prescribing treatment interventions.

### O7 Clinical effectiveness and cost-effectiveness of foot orthoses for people with established rheumatoid arthritis: an exploratory clinical trial

#### Keith Rome^1^, Heidi Clark^2^, Joanna Gray^3^, Peter McMeekin^3^, Michael Plant^4^, John Dixon^5^

##### ^1^AUT University, Auckland, New Zealand; ^2^Podiatry Department, South Tees Hospitals NHS; Foundation Trust, Middlesbrough, UK; ^3^Department of Public Health and Wellbeing, Northumbria University, Newcastle, UK; ^4^Rheumatology Department, South Tees Hospitals NHS Foundation Trust, Middlesbrough, UK; ^5^Health and Social Care Institute, Teesside University, Middlesbrough, UK

###### **Correspondence:** Keith Rome

This abstract is not included here as it has already been published.

### O8 Does the addition of exercise improve the diagnostic accuracy of the ankle-brachial index in people with and without diabetes?

#### Peta Tehan^1,2^, Vivienne Chuter^1,2^, Alex Barwick^3^, Mathew Sebastian^4^

##### ^1^Hunter Medical Research Institute, New Lambton, Australia; ^2^University of Newcastle, Ourimbah, Australia; ^3^Southern Cross University, Gold Coast, Australia; ^4^Vascular Health Care, Gateshead, Australia

###### **Correspondence:** Peta Tehan

**Objectives**

The ankle-brachial index (ABI) is used to identify peripheral arterial disease (PAD) and is regularly employed by podiatrists. The postexercise ABI is an additional diagnostic test. The aim of this study was to determine diagnostic accuracy of resting and postexercise ABI for detecting PAD in people with and without diabetes.

**Method**

Resting ABI and postexercise ABI measurements were extracted from medical records of patients referred to a vascular laboratory for investigations due to suspected PAD. Diagnostic accuracy was determined and compared using colour duplex ultrasound (CFDU) using sensitivity, specificity and receiver operating curve (ROC). Data was also divided into sub-groups by disease severity, and disease location, and diagnostic accuracy was then calculated for these groups.

**Results**

206 limbs were included, 83 with diabetes and 123 without diabetes. Postexercise ABI had the highest sensitivity overall in people with diabetes (61.43%) and without diabetes (66.99%) compared to the resting ABI which had lower sensitivity in people with diabetes (39.44%) and those without diabetes (46.08%). Sub-group analysis demonstrated that in more proximally located PAD, the postexercise ABI had highest sensitivity in people with (100%) and without diabetes (80%). ROC analysis showed that overall the resting ABI had the highest AUC overall in people with (0.74) and without diabetes (0.75).

**Conclusions**

Whilst the addition of exercise improved the sensitivity of ABI in both people with and without diabetes, the overall accuracy of ABI and postexercise ABI was low in both populations, with resting ABI yielding higher accuracy compared to postxercise ABI. The postexercise ABI may be of limited additional clinical value.

### O9 First metatarsophalangeal joint (MTPJ) mobility assessment - consistency between subjective ‘feel’ and quantitative quasi-stiffness

#### Marabelle Heng^1^, Pui Wah Kong^2^

##### ^1^Singapore General Hospital, Singapore, Singapore; ^2^Nanyang Technological University, National Institute of Education, Singapore, Singapore

###### **Correspondence:** Marabelle Heng

**Objectives**

The first metatarsophalangeal joint (MTPJ) mobility is assessed in patients with hallux conditions or apropulsive gait. In subjective clinical assessment, the tester rates the joint as “hypermobile”, “normal” or “stiff”. This study examined the consistency between subjective “feel” and quantitative quasi- stiffness in the first MTPJ.

**Method**

18 healthy participants with no reported foot problems or joint disorders were assessed. The first MTPJ mobility of participants were subjectively rated by an experienced podiatrist as “hypermobile”, “normal” or “stiff”. Next, the first MTPJ quasi-stiffness was determined (in Nmm/degree) through force- displacement measurements. The quasi-stiffness were measured twice with values averaged to minimise random error. To check the consistency between subjective rating and quantitative measurement, quasi-stiffness values were compared between the subjectively classified groups - “hypermobile” vs. “normal”- using Mann Whitney U test (since no participants were rated “stiff”).

**Results**

Data of 6 participants were excluded from analysis due to technical fault. For the remaining 12 participants, 6 were subjectively rated “hypermobile” while 6 were rated “normal”. The “normal” group has significantly higher median (IQR) quasi-stiffness [15.42 (22.01) Nmm/degree] than “hypermobile” group [8.56 (3.69) Nmm/degree, p = .016]. This suggests that subjective classification of first MTPJ mobility corresponds well with objective quantification of joint quasi-stiffness.

**Conclusions**

There is consistency between subjective rating by experienced tester and quantitative quasi-stiffness. Objective assessment (quasi-stiffness) instead of subjective “feel” is a potential clinical advancement as objective scores may guide choice of orthotic material stiffness and provide useful information on hallux conditions associated with hypermobility (eg. Hallux valgus).

### O10 Are ultrasound features at the first metatarsophalangeal joint associated with clinically- assessed structure and function? A study of people with gout, asymptomatic hyperuricaemia and normouricaemia

#### Sarah Stewart^1^, Nicola Dalbeth^2,5^, Alain C. Vandal^1,3^, Bruce Allen^4^, Rhian Miranda^5^, Keith Rome^1^

##### ^1^Auckland University of Technology, Auckland, New Zealand; ^2^The University of Auckland, Auckland, New Zealand; ^3^Counties Manukau Health, Auckland, New Zealand; ^4^Horizon Radiology, Auckland, New Zealand; ^5^Auckland District Health Board, Auckland, New Zealand

###### **Correspondence:** Sarah Stewart

**Objectives**

It is unclear whether ultrasound evidence of crystal deposition, which is present at the first metatarsophalangeal joint (1MTPJ) in people with gout and asymptomatic hyperuricaemia, is related to clinically-evident structure, function and pain. This study aimed to determine the association between 1MTPJ ultrasound features and clinical foot characteristics.

**Method**

A cross-sectional study was undertaken involving participants with gout (n = 21), asymptomatic hyperuricaemia (n = 29) and normouricaemic controls (n = 34). No participant had clinical evidence of inflammatory arthritis at the time of assessment. Four ultrasound features at the 1MTPJ were analysed: double contour sign, tophus, erosion and synovitis. Clinical characteristics included: 1MTPJ pain Visual Analogue Scale (VAS), Manchester Foot Pain and Disability Index (MFPDI), 1MTPJ temperature, 1MTPJ dorsiflexion motion and gait velocity. Mixed regression models were used to determine the associations between ultrasound and clinical characteristics while adjusting for the diagnostic group. Data were analysed at a Bonferroni-adjusted significance level of <0.01.

**Results**

All participants were men with a mean age of 58 years. Participants with gout had a mean (SD) disease duration of 18 (11) years. Eighty-three percent had a history of 1MTPJ acute arthritis. Presence of the double contour sign was associated with higher MFPDI scores (10.1 in those with the feature present vs. 4.8 in those with the feature absent, *P* < 0.001). Tophus presence was associated with higher MFPDI scores (19.8 vs. 5.9, *P* < 0.001), increased temperature (28.6 °C vs. 26.2 °C, *P* = 0.005) and reduced walking velocity (0.88 m/s vs. 1.00 m/s, *P* = 0.001). No associations were observed between synovitis or erosion and clinical characteristics.

**Conclusions**

Ultrasound features of urate deposition at the 1MTPJ were associated with increased overall patient- reported foot pain and disability and increased temperature of the 1MTPJ which reflects the persistent nature of urate crystals in activating inflammation and pain even in the absence of clinical-evidence of acute arthritis.

### O11 The effect of a cadence retraining protocol on lower limb sagittal plane kinematics and EMG activity in a normal population of social runners

#### Christopher Maher, Luke Donnan, Paul Tinley

##### Charles Sturt University, Albury, Australia

###### **Correspondence:** Christopher Maher

**Objectives**

Altering cadence is a method of running retraining. Its effect on knee flexion, ankle dorsiflexion, tibialis anterior (TA) and medial gastrocnemius (MG) activation during running are largely unknown. This study aimed to characterise the effect increasing running cadence on ankle dorsiflexion, knee flexion and TA and MG activity.

**Method**

16 runners participated in the study. Subjects were measured for base line parameters and trained to increase their preferred running cadence (PC) by 10%. Kinematic and EMG data were collected for PC, post initial training (PIT) and post three weeks training (PT). PC was remeasured after six weeks.

**Results**

No significant changes occurred in sagittal plane kinematics or MG function. However it was noted there was a 9% decrease in ankle dorsiflexion during loading response (LR) from PC to PT, as well as an 11% decrease during pre-activation. TA activity decreased during LR and pre activation (PA) between PIT and PT. However there was no significant change in TA from PC to PT. There was however a 20% reduction in TA activity from PC to PT during LR, and a 10% decrease during PA for the same time period.

**Conclusions**

This study provides limited evidence increasing cadence reduces TA activity and ankle dorsiflexion during loading response. This has potential as treatment for conditions where increased ankle dorsiflexion and TA activity are contributing factors. However further research with a longer protocol is required to provide stronger empirical evidence on the topic.

### O12 The identification and appraisal of assessment tools used to evaluate metatarsus adductus: a systematic review

#### Nicole Marshall^1^, Emily Ward^1^, Cylie Williams^2^

##### ^1^University of South Australia, South Australia, Australia; ^2^Monash University, Victoria, Australia

###### **Correspondence:** Nicole Marshall

**Objectives**

Metatarsus adductus is the most common congenital foot deformity in newborns. It involves adduction of the metatarsals at the lisfranc joint. A systematic literature review was conducted to investigate the following question: What tools are used to identify and quantify metatarsus adductus and how reliable, valid and sensitive are they?

**Method**

The following databases were searched from inception to June 2016: Medline, EMBASE, CINAHL, Scopus, Wed of Science and AMED. Two researchers initially searched all articles by screening abstracts. If there was any doubt as to an article’s eligibility, the full text paper was retrieved. Reference lists and citations of all retained studies were examined in an attempt to locate further studies. Articles were excluded if they were not in English or had no psychometric properties. Studies included in the review reporting psychometric properties of measurement tools were critically appraised using the COSMIN critical appraisal tool.

**Results**

282 articles were screened at abstract and 25 articles screened from full text. 15 articles were included and appraised using the COSMIN critical appraisal tool. Techniques to measure metatarsus adductus included; the heel bisector method, photocopies, ultrasound, footprints, dynamic foot pressure and radiographs. There was a paucity of quality data reporting the reliability, validity or sensitivity for measuring metatarsus adductus. Several radiographic angles showed good reliability (ICC – 0.84–0.972) in adults during pre-operative planning. However, radiographs are rarely used clinically due to the unnecessary radiation exposure, cost and time required to perform these measures for low acuity interventions.

**Conclusions**

Further psychometric testing is required to determine if the most common non-radiographic measures of metatarsus adductus should be considered acceptable for clinical use.

### O13 Relationships among neuropathies, vascular reactivity and bone in the diabetic foot

#### Alex Barwick^1^, John Tessier^2^, Xanne Janse de Jonge^2^, Vivienne Chuter^2^

##### ^1^Southern Cross University, QLD, Australia; ^2^University of Newcastle, NSW, Australia

###### **Correspondence:** Alex Barwick

This abstract is not included here as it has already been published.

### O14 Clinical characteristics of foot ulceration in people with chronic gout

#### Keith Rome^1^, Kathyrn Erickson^2^, Cynthia Otene^2^, Hazra Sahid^2^, Karyn Sangster^2^, Peter Gow^2^

##### ^1^AUT University, Auckland, New Zealand; ^2^Counties Manukau District Health Board, Auckland, New Zealand

###### **Correspondence:** Keith Rome

This abstract is not included here as it has already been published.

### O15 Flip-flop footwear with a moulded foot-bed for the treatment of foot pain: a randomised controlled trial

#### Vivienne Chuter, Angela Searle, Martin Spink

##### University of Newcastle, Callaghan, NSW, Australia

###### **Correspondence:** Martin Spink

This abstract is not included here as it has already been published.

### O16 Growth trajectories of the paediatric foot: relationships with obesity

#### Stewart Morrison^1^, David McCarthy^2^, Ryan Mahaffey^3^

##### ^1^University of Brighton, Brighton, UK; ^2^London Metropolitan University, London, U; ^3^St Mary’s University, Twickenham, London, UK

###### **Correspondence:** Stewart Morrison

**Objectives**

Determinants of growth of the paediatric foot and limb are multi-factorial. Understanding these determinants is important for recognising factors which impact on foot development. The aim of this study was to determine associations between obesity and paediatric foot dimensions.

**Method**

A retrospective analysis of paediatric foot dimensions (foot length – FL; foot width – FW) in 3,713 children aged 3–18 years was undertaken. BMI was converted to gender-specific Standard Deviation Scores (BMI SDS) and each participant assigned a weight category. Foot length was defined as the measurement from posterior calcaneus to the most distal aspect of the longest toe. Foot width was calculated between the medial first metatarsal head and the lateral fifth metatarsal head. Measurements were taken using modified callipers. Logistic regression was used to determine relationships between foot length and foot width and weight category.

**Results**

Compared with obese peers, typical weight (FL: *p* = ≤ .05, OR .83; FW: *p* = ≤ .05, OR .56) and underweight (FL: *p* = ≤ .05, OR .76; FW: *p* = ≤ .05, OR .41) boys had significantly shorter and narrower feet. Overweight (FL: *p* = .02, OR .88; FW: *p* = .02, OR .72), typical weight (FL: *p* = ≤ .05, OR .77; FW: *p* = ≤ .05, OR .47) and underweight (FL: *p* = ≤ .05, OR .70; FW: *p* = ≤ .05, OR .33) girls had significantly shorter and narrower feet.

**Conclusions**

These findings highlight obesity as an important determinant of paediatric foot dimensions. Given the current prevalence of obesity in children and young people, these findings may have population wide implications for paediatric foot health.

### O17 A multi-faceted investigation of non-invasive vascular assessment in people with diabetes

#### Vivienne Chuter^1^, Peta Tehan^1,3^, Jennifer Sonter^2^, Sean Lanting^1^

##### ^1^University of Newcastle, Ourimbah, NSW, Australia; ^2^Western Sydney University, Campelltown, NSW, Australia; ^3^Priority Research Centre for Generational Ageing, Hunter Medical Research Institute, New Lambton, NSW, Australia

###### **Correspondence:** Peta Tehan

This abstract is not included here as it has already been published.

### O18 Foot and ankle characteristics associated with falls in people with rheumatoid arthritis: a prospective longitudinal study

#### Angela Brenton-Rule^1^, Nicola Dalbeth^2^, Hylton B. Menz^3^, Sandra Bassett^1^, Keith Rome^1^

##### ^1^AUT University, Auckland, New Zealand; ^2^Univeristy of Auckland, Auckland, New Zealand; ^3^La Trobe University, Melbourne, Australia

###### **Correspondence:** Angela Brenton-Rule

**Objectives**

People with rheumatoid arthritis (RA) have an increased risk of falls. Previous studies have identified that foot and ankle problems are associated with falls in older adults. The aim of this prospective observational study was to determine whether foot and ankle characteristics are associated with falls in people with RA.

**Method**

Adults with RA were recruited from rheumatology outpatient clinics in Auckland, New Zealand. RA characteristics, common fall risk factors, and foot and ankle variables were measured at baseline. Participants were then followed for 12 months, to record the occurrence of falls, using monthly falls calendars and telephone calls. Univariate parametric and non-parametric analysis compared fallers and non-fallers on baseline variables to determine significant differences. Logistic regression analysis, including age and all baseline variables at a level of *p* < 0.15 in univariate analysis but excluding 12- month fall history, identified baseline variables which were independent predictors of falls over the 12- month period.

**Results**

201 participants completed the baseline assessment and 196 (98%) completed follow-up to 12 months. Eighty-four (42%) participants fell at least once and 39 (19%) experienced multiple falls over the 12-month follow-up period. Fallers had significantly higher tender joint count, increased medications, were more likely to take psychotropic medication or use an assistive device, had increased eyes-closed postural sway and were more likely to have tender foot or ankle joints and a 12-month fall history. In logistic regression analysis, psychotropic medication (OR ratio 2.4, *p* = 0.03) and presence of foot or ankle tender joints (OR 2.0, *p =* 0.03) were independent predictors of falls.

**Conclusions**

Psychotropic medications and tender joints in the feet and ankles are independent predictors of falls in people with RA. Clinical assessment of synovitis in the feet and review of psychotropic medications may be of benefit when considering falls prevention in people with RA.

### O19 Maximising falls related injury prevention opportunities for our older clients

#### Kristy Robson^1^, Julia Coyle^1^, Rodney Pope^2^

##### ^1^Charles Sturt University, Albury, NSW, Australia; ^2^Bond University, Gold Coast, QLD, Australia

###### **Correspondence:** Kristy Robson

**Objectives**

Fall related injury rates in older Australians continue to rise despite concerted efforts to manage fall risks. Understanding fall risk from the perspective of the older person may provide valuable knowledge that can assist podiatrists with falls prevention. The study aim was to understand regional older people’s experiences of falling.

**Method**

A qualitative approach using hermeneutics was used to explore the perceptions of older people towards the risk of falling. Hermeneutics arises from an interpretive theoretical viewpoint, where the main goal is to understand human experience and actions. The study involved 33 participants residing in southern NSW who took part in semi-structured focus groups and semi-structured in-depth interviews. Interviews were audio-recorded, transcribed verbatim and individually and collectively analysed to identify key themes.

**Results**

The findings suggest that limited dialogue between older people and health professionals was occurring with the participants on their falls history and individual falls related risks. This was especially evident in less frail older populations. As such, it was not until a significant fall event occurred that falls prevention strategies were instigated. In addition, participants indicated that they were reluctant to initiate discussion with health professionals on falling, particularly when it was not the main purpose of the visit. These themes indicate that health professionals may be missing opportunities to instigate early falls prevention strategies with their older clients.

**Conclusions**

Podiatrists are in a unique position to be contributing to falls prevention given the nature and demographic of practice. Importantly, podiatrists need to start the conversation with older clients about their mobility concerns so that early falls education and intervention strategies can be instigated to minimise their risk of falling.

### O20 Repeatability and feasibility of plantar pressure analysis in people following stroke

#### Stewart Morrison^1^, Alison Rogers^2,3^, Terry Gorst^4^, Joanne Paton^4^, Jenny Freeman^4^, Jon Marsden^4^, Mary Cramp^5^

##### ^1^University of Brighton, Brighton, UK; ^2^University of East London, London, UK; ^3^Keele University, Keele, UK; ^4^Plymouth University, Plymouth, UK; ^5^University of the West of England, Bristol, UK

###### **Correspondence:** Stewart Morrison

**Objectives**

Clinical evaluation of foot function following stroke is important. This study aimed to determine repeatability and feasibility of plantar pressure analysis, and was part of a larger project (FAiMiS) which investigated foot and ankle predictors of mobility and balance in people following stroke.

**Method**

Fourteen participants (mean age 60.8 ± 9.2 years; time since stroke 60.1 ± 56.1 months; eight Right- side) were recruited from local stroke groups and NHS clinics, along with 14 healthy controls (mean age 62.5 ± 13.19 years; 12 Right dominant). Plantar pressure variables were measured using a high resolution pressure mat (Tekscan^(R)^ HR Mat) sampling at 50Hz. A two-step protocol was used and each participant walked at self-selected walking speed. Three walking trials were collected on two occasions. Geometric masks were compared for four and eight regions Repeatability was explored with Intraclass correlation coefficients (3,1) and independent t-tests assessed between-group comparisons.

**Results**

Artefact-free data was gathered in all participants. Peak pressure and contact area demonstrated excellent repeatability for most plantar foot regions in the four-region model (ICC ≥ 0.82), and moderate repeatability for peak pressure and contact area at the toes (ICC 0.76 and 0.58 respectively). The eight-region analysis demonstrated a broader range of scores (ICC 0.36–0.98), with moderate repeatability for loading at medial toes (ICC 0.65) and poor reliability (ICC 0.36) for medial forefoot. There were significant differences (*p* ≤ .05) in peak plantar pressure and contact area between the groups for all of the 4 foot regions.

**Conclusions**

Use of the four region mask in this study was feasible to implement and yielded data with moderate to excellent repeatability. Our protocol for the clinical evaluation of the foot may hold relevance for clinical practice.

### O21 Dealing with the death of a long term patient; what is the impact and how do podiatrists cope?

#### Kristy Robson^1^, Cylie Williams^2^

##### ^1^Charles Sturt University, Albury NSW, Australia; ^2^Monash University, Melbourne, VIC, Australia

###### **Correspondence:** Kristy Robson

**Objectives**

It is common for long-term professional relationships to develop between podiatrists and patients. Patient’s decline in health or death may impact a practitioner’s mental wellbeing. The aim of this project was to understand the impact of long term patient death on podiatrists and identify coping strategies.

**Method**

Interpretative phenomenological analysis was used to explore the perceptions of podiatrists on the personal and professional impact following the death of a long term patient. Individual semi-structured interviews were conducted with podiatrists across Australia. Inclusion criteria was that the podiatrist must have been practicing longer than 5 years and who experienced a long term patient die in the previous 12 months. General demographics of recency of practice, gender, state, Brief Resilience Scale (BRS) and the Abbreviated Maslach Burnout Inventory (MBI) were collected. Interviews were audio-recorded, transcribed verbatim and individually analysed to identify key themes.

**Results**

Fifteen podiatrists (11 female) with a median of 15 (range 8–50) year’s experience participated. The mean(SD) BSI was 3.48 (0.94), two reported MBI scores indicative of emotional exhaustion, no depersonalisation and all scores indicated personal accomplishment.

Three major themes emerged: acknowledging connections, willing to share and listen, and creating support through starting the conversation. Participants indicated importance in recognition of the emotional influence of professional-patient relationships. They also discussed the importance of debriefing about death with the right person, which was most commonly colleagues. Participants talked about the emotional impact of death, suggesting need for supporting discussion and resources, especially for new graduates.

**Conclusions**

Death and dying can be an emotive topic and one which podiatrists may not be prepared for, yet likely to have to deal. These findings enable a better understanding of the impact of patient death and provide possible future directions for the profession to better support podiatrists in this area.

### O22 The hidden risk factors for diabetes-related lower limb amputations

#### Adrian Singh^1,2^, Peter Lazzarini^3,4^, Lloyd Reed^4^, Gavin Turrell^2,5^

##### ^1^Institute for Urban Indigenous Health, Brisbane, Queensland, Australia; ^2^School of Public Health and Social Work, Queensland University of Technology, Brisbane, Queensland, Australia; ^3^Allied Health Research Collaborative, Metro North Hospital & Health Service, Brisbane, Queensland, Australia; ^4^School of Clinical Sciences, Queensland University of Technology, Brisbane, Queensland, Australia; ^5^Institute of Health and Ageing, Melbourne, Victoria, Australia

###### **Correspondence:** Adrian Singh

**Objectives**

Social determinant factors - in socioeconomic status (SES), geographical remoteness (GR) and Aboriginal Torres Islander status (ATSI) - are key drivers of poor health outcomes in Australia. No studies have investigated the association between multiple social determinant factors and diabetic foot disease. Thethis study will investigate associations between multiple social determinant factors and amputations.

**Method**

This study was a retrospective analysis of data obtained on all patients hospitalised with diabetic foot disease in Queensland between 2004–2011 from the Queensland hospital discharge database. Age, sex, SES, GR, ATSI status, diabetic foot disease disorders and amputation procedures were obtained using ICD codes for each hospital admission. Logistic regression were undertaken to analyse associations between these variables and amputation.

**Results**

Overall, 19,790 patients were hospitalised with diabetic foot disease and 4,442 (22.4%) underwent an amputation procedure. Multivariate analysis identified only indigenous people were independently associated (OR 1.63 [95% CI 1.23–2.16])) with amputation after adjusting for all other variables (p < 0.05).

**Conclusions**

Findings indicate that ATSI people with diabetic foot disease are at higher risk of amputation than non-indigenous people, even after controlling for geographical remoteness and socioeconomic status. More research is required to investigate whether individual level social determinant factors may impact on this disparity.

### O23 Association between ankle equinus and plantar pressures in people with diabetes. A systematic review and meta-analysis

#### Angela Searle, Martin Spink, Alan Ho, Vivienne Chuter

##### University of Newcastle, NSW, Australia

###### **Correspondence:** Martin Spink

**Objectives**

Diabetes related restriction in ankle joint range of dorsiflexion is proposed to contribute to elevated plantar pressures implicated in the development of foot ulcers. The aim of this review was to investigate the evidence of an association between limited ankle joint dorsiflexion and plantar pressures in people with diabetes.

**Method**

A systematic search of EBSCO Megafile Premier (containing MEDLINE, CINAHL, SPORTSdiscus, Academic Search Complete) and The Cochrane Library was conducted to 23rd November 2016. Two authors independently reviewed and selected relevant studies. Included studies investigated ankle dorsiflexion range of motion and plantar pressures in people with diabetes. Studies were excluded where the individuals had current plantar foot ulcers, neurologically induced limited ankle joint range of motion (e.g. stroke or cerebral palsy), or the studies reported ground reaction forces or joint moments only, or the data could not be obtained.

**Results**

Fifteen studies met the inclusion criteria. Three studies in the meta-analysis found that equinus increases plantar pressures with a small, but significant effect size (ES = 0.26, CI 95% 0.11 to 0.41, p = 0.001). Of the remaining twelve studies, eight found evidence of an association between limited ankle dorsiflexion and increased plantar pressures while four studies found no relationship. Limited ankle dorsiflexion and increased plantar pressures were found in all studies where the sample population had a history of neuropathic foot ulceration. The same association was not found in studies where the population had neuropathy and no history of foot ulcer.

**Conclusions**

Limited ankle joint dorsiflexion may contribute to elevating plantar pressures in people with diabetes. An equinus may be an early clinical indicator of increased ulcer risk, and it would be advisable for clinicians to assess for this movement restriction, especially in high risk groups such as those with neuropathy.

### O24 Podiatry screening of the Orthopeadic Access Service (OAS) to reduce outpatient wait list, a 10 year retrospective

#### Nicole Spooner, Peter Schoch

##### Barwon Health, Geelong, Victoria, Australia

###### **Correspondence:** Nicole Spooner

**Objectives**

In 2005, Barwon Health Orthopaedic outpatients had over 1200 patients waiting for a first appointment. This included a large number of patients with foot and ankle problems. Time from referral to initial appointment with the surgeon averaged 2.5 years.

**Method**

Following the successful introduction of physiotherapy led screening clinics for people with shoulder, knee and spinal problems, the orthopaedic outpatient wait list was audited to identify patients with foot/ankle problems who could be suitable for an assessment with the podiatrist. The Orthopaedic Assess Service (OAS) foot and ankle clinic was established. Podiatrists assessed patients who had been triaged as having non or semi urgent problems, to determine who required a surgical consult and who could be managed via conservative measures. Patients were then referred on to see a surgeon, if indicated, and/or referred to allied health services for conservative management.

**Results**

Since the introduction of the OAS foot and ankle clinic, waiting times for initial appointment for foot and ankle patients with non-urgent problems have decreased to an average of 4.5 months. This reduction of waiting time has been maintained over the last decade. Up to 40% of patients assessed by the podiatrist are discharged to conservative management without having to see a surgeon. Patient satisfaction with the OAS is high and the majority of patients are happy to initially be assessed by a podiatrist.

**Conclusions**

A podiatry led screening clinic can work collaboratively and successfully with the orthopaedic unit to help patients with foot and ankle problems access the most appropriate care and contribute to reducing waiting times for service.

### O25 Non-invasive lower limb small arterial measures co-segregate strongly with foot complications in people with diabetes

#### Sean Lanting^1^, Stephen Twigg^2^, Nathan Johnson^2,3^, Michael Baker^4^, Ian Caterson^5^, Vivienne Chuter^1,6^

##### ^1^School of Health Sciences, University of Newcastle, Ourimbah, Australia; ^2^Charles Perkins Centre, University of Sydney, Sydney, Australia; ^3^Discipline of Exercise and Sport Science, University of Sydney, Lidcombe, Australia; ^4^School of Exercise Science, Australian Catholic University, Strathfield, Australia; ^5^Boden Institute of Obesity, Nutrition, Exercise and Eating Disorders, University of Sydney, Sydney, Australia; ^6^Priority Research Centre for Physical activity and Nutrition, University of Newcastle, Callaghan, Australia

###### **Correspondence:** Sean Lanting

**Objectives**

It is unclear how well non-invasive lower-limb vascular assessments can identify those at risk of foot complications in people with diabetes. We aimed to investigate the relationship between a history of foot complication (ulceration or amputation) and non-invasive vascular assessments in people with diabetes.

**Method**

Bilateral ankle brachial index (ABI), toe brachial index (TBI) and continuous wave Doppler (CWD) were performed in 127 adults with diabetes (97% type 2; age 66.08 ± 11.4 years; 55% men; diabetes duration 8.8 ± 7.6 years; 28% on insulin therapy; 31% with foot complication history). Correlations were performed between known risk factors for, and documented history of, foot complications. Regression analysis was used to determine the effect of TBI on the likelihood of a prior foot complication.

**Results**

History of foot complication and presence of neuropathy were strongly correlated (r = 0.572, p < 0.01). Moderate correlations with history of foot complication were found for a TBI of <0.6 (r = 0.451, p < 0.01), diabetes duration (r = 0.333, p < 0.01) and the most recently documented HbA1c (r = 0.295,

p < 0.05). Correlations between history of foot complication and ABI (r = 0.153, p = ns) and CWD (r = 0.124, p = ns) were weak and non-significant. By logistic regression, the likelihood of foot complication history was higher for a TBI <0.6 (OR = 7.74, p = 0.001) than longer diabetes duration (OR = 1.06, p = 0.05). HbA1c did not independently predict history of foot complication (OR = 1.10, p = 0.356).

**Conclusions**

Likelihood of foot complication in this population was ~8 times higher when TBI was <0.6. Such clinical risk profiling was not shown by other non-invasive vascular measures. Prioritising the TBI as a measure of lower limb vascular function may be useful to identify those at risk of diabetic foot complications.

### O26 Intentions to use “smart” insole technology in regionally-based adults with diabetes: a cross sectional study

#### Emma Macdonald^1,2^, Byron Perrin^1^, Michael Kingsley^1^

##### ^1^La Trobe Rural Health School, College of Science, Health and Engineering, La Tobe University, Bendigo, Australia; ^2^Diabetes Centre, Goulburn Valley Health, Shepparton, Australia

###### **Correspondence:** Emma Macdonald

**Objectives**

Smart insole technology providing foot health biofeedback might support foot-care in adults with diabetes. However, the level of acceptance and behavioural intention is unclear for this subpopulation, particularly in regional Australia. The aim of this study was to investigate if adults with diabetes would accept and use smart insoles.

**Method**

The validated Unified Theory of Acceptance in Technology (UTAUT) questionnaire was used in a sample of adults with diabetes from regional Australia. The UTUAT comprises 7 psychosocial factors related to behavioural intention to use technology, such as a smart insole. These factors include aspects relating to performance expectancy, effort expectancy, attitude, social influence, facilitating conditions, self-efficacy and anxiety. Correlation analysis was used to explore the relationship between these factors and behavioural intention to adopt smart insole technology. Independent t- tests and ANOVAs were used to explore differences in behavioural intention across demographic and diabetes-related factors.

**Results**

Fifty-three adults with a mean age 61 ± 12 years (M ± SD) completed the questionnaire, with 68% being male and 75% type 2 diabetes with a mean duration of 17.3 ± 10.7 years. Positive correlations existed between behavioural intention and the following: attitude (r = 0.5, p < 0.001), social influence (r = 0.4, p = 0.010), facilitating conditions (r = 0.4, p = 0.004), self-efficacy (r = 0.5, p < 0.001), and a negative correlation was found between behavioural intention and anxiety (r = −0.42, p = 0.003). There was no significant difference in behavioural intention with respect to age, diabetes duration and education. Patients with a history of foot ulceration had higher behavioural intention scores than those that did not (t = 2.7, p = 0.010).

**Conclusions**

Patients were generally positive about adopting a smart-insole for foot-health monitoring. Key psychosocial factors were associated with patients’ intention to use biofeedback technology, which should be considered when implementing new technology within a preventative health plan.

### O27 A longitudinal evaluation of site specific plantar pressures in people with diabetes related foot ulcers and diabetes controls without ulcers

#### Malindu Fernando^1,7^, Robert Crowther^2,8^, Peter Lazzarini^4,5^, Saiumaeswar Yogakanthi^10^, Kunwarjit Sangla^3^, Petra Buttner^9^, Rhondda Jones^11^, Jonathan Golledge^1,6^

##### ^1^Vascular Biology Unit, Queensland Research Centre for Peripheral Vascular Disease, College of Medicine and Dentistry, James Cook University, Townsville, Queensland, Australia; ^2^Sports and Exercise, School of Health and Wellbeing, University of Southern Queensland, Ipswich, Queensland, Australia; ^3^Department of Diabetes and Endocrinology, The Townsville Hospital, Townsville, Queensland, Australia; ^4^Allied Health Research Collaborative, Metro North Hospital & Health Service, Queensland Health, Brisbane, Queensland, Australia; ^5^School of Clinical Sciences, Queensland University of Technology, Brisbane, Queensland, Australia; ^6^Department of Vascular and Endovascular Surgery, The Townsville Hospital, Townsville, Queensland, Australia; ^7^Podiatry Service, Townsville Community Health Service, Townsville, Queensland, Australia; ^8^Movement Analysis Laboratory, Sports and Exercise Science, James Cook University, Townsville, Queensland, Australia; ^9^Centre for Chronic Disease Prevention, James Cook University, Cairns, Queensland, Australia; ^10^Faculty of Medicine, Nursing and Health Science, Monash University, Melbourne, Victoria, Australia; ^11^Australian Institute of Tropical Health and Medicine, James Cook University, Townsville, Queensland, Australia

###### **Correspondence:** Malindu Fernando

**Objectives**

Little is known about plantar pressures during ulcer healing in people with diabetes related foot ulcers. The aim of this study was to investigate plantar pressures at baseline, three months and six months in cases with foot ulcers compared to diabetes controls without ulcers.

**Method**

Standardised protocols were used to measure mean peak plantar pressure and pressure-time integral at 10 plantar foot sites (the hallux, lesser toes, metatarsals 1 to 5, mid-foot, medial heel and lateral heel) during barefoot walking. Measurements were performed at three study visits: baseline, three and six months. Linear mixed effects random-intercept models were utilised to assess whether plantar pressures differed between cases and controls after adjusting for age, sex, body mass index, neuropathy status and follow-up time. Standardised mean differences (Cohen’s d) were used to measure effect size.

**Results**

Twenty-one cases and 69 controls started the study and 16 cases and 63 controls completed the study. Cases had a higher mean peak plantar pressure at several foot sites including the lesser toes (p = 0.005, Cohen’s d =0.36), and mid foot (p = 0.01, d = 0.36) and a higher pressure-time integral at the hallux (p < 0.001, d = 0.42), metatarsal 1 (p = 0.02, d = 0.33) and mid foot (p = 0.04, d = 0.64) compared to controls at baseline and follow-up. During follow-up, both cases and controls demonstrated a reduction in pressure-time integral at these specific sites (p < 0.05).

**Conclusions**

Plantar pressures during gait were higher in patients with foot ulcers compared to patients without foot ulcers throughout follow-up despite gradual reductions over time. These results support on-going offloading in people with chronic DFUs.

### O28 Depression, anxiety and stress in people with and without plantar heel pain: a cross-sectional observational study

#### Matthew Cotchett, Shannon Munteanu, Karl Landorf

##### La Trobe University, Bendigo, Australia

###### **Correspondence:** Matthew Cotchett

This abstract is not included here as it has already been published.

### O29 Improving clinician access to prescribing scheduled medicines - what the evidence shows

#### Paul Bennett^1,2^, Anthony Short^1,3^, Antonio Cuesta-Vargas^4,5^

##### ^1^Queensland University ofTechology, Brisbane, Qld, Australia; ^2^Institute of Health and Biomedical Innovation, Brisbane, Qld, Australia; ^3^The Podiatry Practice, Brisbane, Qld, Australia; ^4^Universidad de Malaga, Andalucía, Spain; ^5^Instituto de Investigacion Biomédica de Malaga, Andalucia, Spain

###### **Correspondence:** Paul Bennett

**Objectives**

Prescription of scheduled medicines represents an extended scope of practice for podiatrists and was approved by the Australian Health Workforce Ministerial Council in 2010. To date only 74 (1.6%) of Australia’s 4,655 podiatrists have gained endorsement^1^. This paper explores barriers and enablers to gaining qualifications to prescribe.

**Method**

A cross sectional study design was undertaken in 12 clinical locations across three states of Australia where active prescribing to management foot and ankle conditions took place. Public and private hospital settings delivering orthopaedic, vascular, rheumatology and podiatry services and several private practice and university clinic locations were surveyed in the study. Service providers included podiatrists with endorsement to prescribe scheduled medicines, podiatric surgeons and specialist medical practitioners. Data was captured over a two month period during 2016. All prescriptions were verified as meeting the criteria for inclusion according to the Podiatry Board of Australia’s list of scheduled medicines.

**Results**

Approximately 300 patients were screened of which 40 received active therapeutic intervention. A total of 82 medications were prescribed. The most commonly prescribed medications, excluding local anaesthetic injections included; analgesics (33%), antibiotics (18%), NSAID’s (15%), glucocorticosteroid (14%), antifungal and conscious sedation agents (4%). 80% of these medications were schedule 4 with the remainder in either schedule 2 or 3. 68% of patients received 2 or more medications during an episode of care, usually associated with a surgical service. Hospital and university based outpatient clinics prescribed significantly few “multiple medications” during an episode of care.

**Conclusions**

Barriers to gaining adequate clinical experience include; lack of easy access to suitable training facilities, inadequate number of available clinical supervisors and inefficient/inordinate time demands. Based on these findings, an innovative model of clinical training is proposed to enable interested and motivated podiatrists to gain access to these new skills.

### O30 Application of ‘fast cast’ and ‘needle tenotomy’ protocols with the Ponseti method for improved clubfoot management in Bangladesh

#### Angela Evans^1,3^, Mamun Chowdhury^1,2^, Sohel Rana^2^, Abu Hena Mahboob^2^

##### ^1^Walk for Life, Dhaka, Bangladesh; ^2^Orthopaedics, MMCH, Mymensingh, Bangladesh; ^3^La Trobe University, Melbourne, Australia

###### **Correspondence:** Angela Evans

**Objectives**

The management of congenital talipes equino varus (*clubfoot deformity*) has been transformed in the last 20 years, with surgical correction replaced by the non-surgical Ponseti method. The Ponseti method, consists of corrective serial casting followed by maintenance bracing, repeatedly demonstrated to give best results, now regarded as ‘gold standard’ treatment.

**Method**

Using Level 2 evidence, the casting phase of Ponseti method was modified for children aged <1 year. Standard Ponseti method averages six weekly casts, additional three week cast after Achilles tenotomy, then brace phase. On average, constant plaster cast application is nine weeks (63 days).

Modifying the Ponseti protocol, corrective casts were changed every three days, reducing plaster immobilization to approximately 40 days. Tenotomy was performed using a 21-gauge needle, rather than a scalpel blade, an adapted technique found to reduce bleeding.

The adapted Ponseti protocol began June 2015.

Ethical approval from Mymensingh Medical College (MMCH/2015/9413).

**Results**

123 children study participants; 88 male, 35 female. 113/123 cases typical clubfoot, 5 atypical, 5 syndromic. Average age at first cast was 51 days (13–240 days).

The average number of casts was 5 (2–12 casts). The average number of days between first cast and brace was 44.8 days (7–129), with 21 days in post-tenotomy cast. Average corrective casting was 24 days. Achilles tenotomy in 100/123 cases, using the needle technique.

Minor complications in 7 cases - 4 skin lesions, 3 disrupted casting phase. Parents preferred reduced casting, less concerned about unseen skin wounds.

**Conclusions**

‘Fast cast’ protocol was successfully used in infants aged <8 months.

The ‘fast casts’ protocol is an effective adaption of Ponseti method. The main benefits are: reduced plaster immobilization – average 24 days ‘fast casts’ protocol versus 42 days traditional method – more effective casts; less skin wounds; happier parents.

### O31 An investigation of the relationships between footprints, foot strength, joint flexibility, vitamin D and iron levels in children with and without leg pains aged three to 12 years

#### Angela Evans^2^, Trupti Berde^1^, Prajakta Joshi^1^, Nehal Shah^1^, Raju Khubchandani^1^

##### ^1^Department of Pediatrics, Jaslok Hospital and Research Center, Mumbai, India; ^2^La Trobe University, Melbourne, Australia

###### **Correspondence:** Angela Evans

**Objectives**

Growing pains (GP) are long-described, frequent paediatric presentations with prevalence approximating 30%, yet misunderstood and poorly managed. Recent research hypothesises overlapping features of growing pains and Ekbom’s syndrome. This study aims to determine leg pain sub-groups and relationship with foot posture, foot strength, joint hypermobility, vitamin D and iron.

**Method**

Ethical Approval was obtained from Human Ethics Committee, Jaslok Hospital and Research Centre, Mumbai. Informed consent was gained from parents/carers. A case controlled design for children with leg pains and age/gender-matched controls, ages three – 12 years, from Mumbai, India.

Participants were stratified to three leg pain groups (versus control group)

1. GP
2. Ekbom
3. GP/Ekbom’s
4. Controls

Data collected included: demographic, anthropometric, pain scale (Wong-Baker faces), pain questionnaire, joint hypermobility (Beighton scale), foot posture (Arch Index, AI), foot strength (dynamometry), Vitamin D and Fe bloods.

**Results**

Interim results for n = 40, 32 cases: 8 control with mean age 6.92 years (2.6), gender 17 male, 23 female. Significant univariate correlations (Spearman’s *rho*) were obtained between anthropometry, foot strength and features of leg pain viz.

**Table:** 

Waist girth	leg pain sub-type	0.371**
AI	−.0425**	
Height	case vs control	−0.355*
Weight	Ekbom feature	0.381*
GP feature	0.366*	
Eversion	Ekbom feature	0.437**
Plantarflexion	Ekbom feature	0.313**

No significant relationship has been found for leg pains and joint hypermobility, Vitamin D, blood Fe. One participant was anaemic, 9 participants were Vitamin D deficient (0.0–10.0 ng/ml), a further 27 participants were Vitamin D insufficient (11.0–30.0 ng/ml).

**Conclusions**

Paediatric leg pain exists in distinct sub-groups. Preliminary findings from this study show relationship between aspects of anthropometry (ie waist girth, height, weight) and foot strength (ie eversion, plantarflexion) features of paediatric leg pains.

Vitamin D deficiency and insufficiency was very prevalent in this study sample.

### O32 What are the determinants of different forms of professional boundary change for Allied Health Professionals in England and Australia

#### Alison Strode

##### University of Bristol, Bristol, UK

**Objectives**

Health care in England and Australia is currently undergoing reforms responsing to factors such as ageing populations and changes in technology. Both Governments have recognised the need to develop a sustainable health workforce identifying inflexibility of professional boundaries as restricting full utilisation of the skills of the healthcare workforce.

**Method**

This research aimed to identify the determinants of professional boundary change for Allied Health Professions (AHPs) particularly relating to physiotherapy, occupational therapy and podiatry. Using a qualitative methodology, utilising semi-structured interviews, the research examined the views of policy leads, uni and multiprofessional associations and regulators about how professional boundary change had been occurring. Employing an exploratory research approach using two sites, England and Australia, it examined the process that professional boundary change has undergone for AHPs with particular relevance to a subset of AHPs covering therapists namely; occupational therapy, podiatry and physiotherapy.

**Results**

The research identified the forms of boundary change occurring for AHPs in Australia and England and the drivers and constraints contributing to change. The AHP professional status was assessed using Abbott’s (1998) concept that the prestige of a profession is determined by a series of jurisdictional disputes within a system rather than by ambitious strategies orchestrated by the professions themselves. The research showed that professional boundaries for AHPs historically have changed to improve the AHPs’ professional status in both countries however, a number of elements including organisational subordination are producing static boundaries that AHPs need to be aware of.

**Conclusions**

Research concludes that the development of government policy and seizing opportunities have been the strongest determinants in professional boundary change for AHPs in Australia and England. Therefore if AHPs want to improve their professional status, they need to be able to influence policy development and respond to advantageous circumstances.

### O33 Identifying indicators of low health literacy in hospitalised patients

#### Rebecca Jessup^1,3^, Richard Osborne^1^, Alison Beauchamp^1^, Allison Bourne^2,3^, Rachelle Buchbinder^2,3^

##### ^1^Deakin University, Victoria, Australia; ^2^Cabrini Institute, Victoria, Australia; ^3^Monash University, Victoria, Australia

###### **Correspondence:** Rebecca Jessup

**Objectives**

Health literacy is an individual’s ability to find, understand and use health information in order to promote and maintain health. Low health literacy has been found to affect ¼ of all Australians, and 1/3 of individuals presenting to hospitals with foot related complaints.

**Method**

Two cross sectional surveys across two hospitals using the Health Literacy Questionnaire (HLQ) - a 44 item survey covering nine domains: Feeling understood and supported by providers; Having sufficient information to manage my health; Actively managing my health; Social support; Appraisal of health information; Ability to actively engage; Navigating the healthcare system; Ability to find good health information; Understanding health information. Eligibility for both surveys were hospitalisation ≥24 hours in the past 30 days, aged ≥ 18, no cognitive impairment, and discharged home.

**Results**

3121 (33%) individuals participated from the private hospital, and 384 (13%) from the public hospital. Private mean age (SD) 66 (17) years (52% female), public mean age (SD) 64 (17) (49% female). There were significant differences between the overall health literacy of the public hospital and private hospital cohorts for almost all HLQ scales, with much of the difference explained by socioeconomic disparities. Allowing for differences, those who participated in low weekly activity levels, those with depression and/or anxiety, and those with 3 or more chronic conditions reported low health literacy across both hospitals for a number of HLQ domains.

**Conclusions**

Health literacy patterns exist across health behaviours and chronic diseases independent of social and economic determinants of health. Podiatrists working in all healthcare settings may use these patterns to identify individuals at risk of having low health literacy, allowing customisation of health information content and delivery.

### O34 The severity of chronic, disabling foot pain in middle-aged women is correlated with fat mass index and depression

#### Tom Walsh^1,2^, John Arnold^3^, Tiffany Gill^4^, Angela Evans^5^, Alison Yaxley^1^, Catherine Hill^2,4^, E Michael Shanahan^1,2^

##### ^1^Flinders University, Bedford Park, South Australia, Australia; ^2^SA Health, Adelaide, South Australia, Australia; ^3^University of South Australia, Adelaide, South Australia, Australia; ^4^The University of Adelaide, Adelaide, South Australia, Australia; ^5^La Trobe University, Bundoora, Victoria, Australia

###### **Correspondence:** Tom Walsh

**Objectives**

Body composition and poor mental health are risk factors for developing foot pain, but the role of different fat deposits and psychological features related to chronic pain are not well understood. The aim of this study was to investigate the association between body composition, psychological health and disabling foot pain.

**Method**

Eighty-eight women participated in this study: 44 with chronic foot pain (mean (SD) age and BMI of 55.3 (7.0) years and 29.5 (6.7) kg/m^2^) respectively, and 44 age and BMI matched controls. Chronic, disabling foot pain was determined from the Manchester Foot Pain and Disability Index, (foot pain on most days of the week with ≥ one of the functional limitation items for > three months). Body composition was measured using dual x-ray absorptiometry and psychological health (catastrophisation, central sensitization and depression) was measured using three validated questionnaires.

**Results**

Between group analyses found that the risk of chronic, disabling foot pain was not significantly associated with any body composition variable, but was significantly associated with all measures of psychological health. Conversely, the severity of foot pain was significantly correlated with body composition measures: fat mass (total, android, gynoid, visceral), fat mass ratios (visceral / android, visceral / subcutaneous), fat mass index (FMI), and depression. In multivariable analysis, only FMI (β 0.18, 95% CI 0.04–0.31) and depression (β 0.06, 95% CI 0.00–0.12) were correlated with foot pain severity.

**Conclusions**

Psychological health, but not body composition, is associated with the presence of foot pain in women. For women with foot pain, however, FMI is the strongest predictor for severity. Further work is needed to determine if a reduction in FMI reduces the severity of foot pain.

### O35 Quality time: an overstretched high risk foot clinic

#### Christopher Klein^1^, Carol Mioduchowski^2^

##### ^1^Barwon Health, Geelong, Vic, Australia; ^2^University Hospital, Geelong, Vic, Australia

###### **Correspondence:** Christopher Klein

**Objectives**

An Influx of complex patients attending University Hospital Geelong’s Diabetes Foot Unit (DFU) created an increase in pressure in allocated consult times and planned appointments. The aim of this retrospective audit was to quantify this impact on appointment times and assist with further development of the High risk foot service.

**Method**

A retrospective electronic documentation audit was undertaken for the DFU. All the patients attending were referred for diabetes high risk foot management with either an ulcer or charcot.

neuroarthopathy. All patient documentation attending for a specific date range were looked at for a three month period. The data compared fixed/planned appointment times versus actual patient attendance. It also looked at the frequency of attendance in this clinic. It should be noted that did not attend data was omitted from the study.

**Results**

Sixty patients were tracked using the service across the 3 month period showing an average of 2.5 consults were utilised per client. The highest proportion of clients (43%) had a one off visits to the DFU with 2% of the cohort required 8 visits, which was the most visits for a three month period. The number of appointments that were available for this period was 104, this was exceeded with a total of 147 appointments provided.

**Conclusions**

There is an influx in service demand for the high risk foot patient. The current capacity doesn’t match demand. This has an impact on clinician’s ability to spend quality time with patients. Working at 50% above capacity highlights the need for an increase in Geelong’s High Risk Foot services.

### O36 Diabetes education retention: a systematic review

#### Julia Yuncken^1,2^, Cylie Williams^1,2^

##### ^1^Peninsula Health, Frankston, Victoria, Australia; ^2^Monash University, Frankston, Victoria, Australia

###### **Correspondence:** Julia Yuncken

**Objectives**

Diabetes foot education is regularly provided by podiatrists during consults, however little is known about the best method to provide education or how it is retained by patients over time. This systematic review aims to determine how diabetes education is provided and how patients retain that information over time.

**Method**

A literature search was conducted during 2016 of MEDLINE, Science Direct, EMBASE, Web of Science, Cochrane. Once articles had been complied references of included articles were searched for further appropriate articles. These articles were then compared to the inclusion criteria - adult patients diagnosed with diabetes (Type 1 or 2), diabetes educational programs delivered to patients and articles in English. In total 809 articles were sourced from the online and references searches, of these only 34 fit the inclusion criteria.

**Results**

There were no articles included that dealt with foot or podiatry specific education. Included articles were largely based on general diabetes education. Timelines for education retention ranged from 3 months to 5 years. Many of the articles found that over time patients were unable to recall diabetes education provided to them. It was also noted that although some patients could retain the education provided it did not always equate to a change in behaviour eg monitoring blood glucose levels daily or a decrease in foot related behaviours.

**Conclusions**

Currently there is little evidence regarding foot specific diabetes education and how this is retained over time. This review highlights the need for further research into how general diabetes education and foot education is presented to patients and how it affects patient outcomes.

### O37 Evidence based non-surgical treatment options for Morton’s Neuroma

#### Barry Matthews^1^, Sheree Hurn^1,2^, Michael Harding^3^, Rachel Henry^4^, Robert Ware^5^

##### ^1^School of Clinical Sciences, Queensland University of Technology, Kelvin Grove, Brisbane, QLD, Australia; ^2^Institute of Health and Biomedical Innovation, Queensland University of Technology, Kelvin Grove, Brisbane, QLD, Australia; ^3^School of Health Sciences, University of South Australia, Adelaide, SA, Australia; ^4^Rachel Henry Podiatry, Clayfield, Brisbane, QLD, Australia; ^5^Menzies Health Institute Queensland, Griffith University, Nathan, Brisbane, QLD, Australia

###### **Correspondence:** Barry Matthews

**Objectives**

Morton’s Neuroma is a common cause of neuralgia which affects the forefoot impacting weight- bearing activities. A 2004 Cochrane review concluded there was little evidence to support insoles as an intervention. The aim of this study was to conduct a systematic review of the current non-surgical interventions for Morton’s Neuroma.

**Method**

A systematic review was registered with Prospero (registration number CRD42016037405) and the search question yielded 1687 papers from 4 biomedical databases. The title and abstract and full text screen reduced the included papers to 19. A quality bias assessment using the Downs and Black checklist is currently being completed and data will be extracted from the higher quality papers for data synthesis. This process will be completed by the end of 2016. Included in our definition of non- surgical interventions were skin penetration treatments that don’t involve an incision, such as an injection or skin penetrating probe (Macquarie Dictionary surgery definition).

**Results**

The 19 papers from the full text screen includes 7 different non-surgical interventions including: manipulation and mobilisation, ultrasound-guided or electro-stimulator or palpation only alcohol sclerosing therapy, ultrasound-guided cryogenic neuroablation, ultrasound-guided corticosteroid injection, footwear modification and orthoses, botulinum toxin A injection and ultrasound-guided pulsed radio frequency ablation. Included study types from the full text screen range from type II randomised controlled trials to type IV case series (NHMRC evidence hierarchy for intervention studies).

**Conclusions**

The review to date has identified a continuing lack of high quality evidence for most interventions reviewed. Registered podiatrists have an obligation to treat patients according to the principles of evidence-based medicine and this systematic review will help inform a podiatrist’s shared decision making with their patients.

### O38 High risk foot rotations for podiatry staff: outcomes of this experience for podiatrists working across the continuum of care

#### Carol Mioduchowski, Chris Klein, Nicole Spooner, Denuwan Weerakkody

##### Barwon Health, Geelong, Australia

###### **Correspondence:** Carol Mioduchowski

**Objectives**

Seventeen clinicians undertook the opportunity to undergo a structured four week rotation within the Diabetes Foot Unit (DFU) at University Hospital Geelong. Clinicians completed a pre and post survey, used as a measure of improvements in clinical knowledge and confidence in the management of the diabetes high risk foot.

**Method**

A structured four week DFU rotation was developed which included clear learning objectives. A pre and post rotation survey was implimented which measured nineteen knowledge and nineteen confidence domains using a 10 point self-rating scale. Follow up qualitative narrative was also collected about this process and experience. The information was then analysed to look at the outcome of this rotation.

**Results**

The results indicated a significant positive increase in both knowledge and confidence in working with the high risk foot setting with 100% of the participants finding it a very beneficial process in skill building. Further outcomes of this rotation, along with the increase in the knowledge and confidence levels included an augmented awareness of the roles of the multidisciplinary medical team, referral pathways and complex wound management planning.

**Conclusions**

A structured rotation for podiatrists within a high risk foot clinic is a beneficial process to improve knowledge and confidence in managing this. The skills gained can be translated into a range of clinical settings. It improves capability, referral and management pathways of the podiatry workforce within our organisation.

### O39 Understanding the provision and retention of podiatry specific diabetes education: 6 months on

#### Julia Yuncken^1,2^, Cylie Williams^1,2^, Rene Stolwyck^2^, Terry Haines^2,3^

##### ^1^Peninsula Health, Frankston, Victoria, Australia; ^2^Monash University, Frankston, Victoria, Australia; ^3^Monash Health, Cheltenham, Victoria, Australia

###### **Correspondence:** Julia Yuncken

**Objectives**

Health literacy is fundamental to the provision of education within the health care setting. Poor health literacy regularly affects people’s health and their ability to self care. Podiatrists currently provide diabetes specific education to their patients, however little is known how that information is retained over time.

**Method**

This project was embedded within a prospective cohort study with two groups, three podiatrists and 24 clients. Participants were eligible to participate if they were a podiatrist at Peninsula Health or a client who attended podiatry consultations and had diabetes. Data collection included the Problem Areas in Diabetes Questionnaire (PAID), Montreal Cognitive Assessment (MoCA), information covered during the consultation and method of delivery and perceived key educational message from podiatrist and client perspectives at the time of the appointment and 6 months post appointment.

**Results**

There were 24 client participants included in this study with an average duration of diabetes 15 (11.8), mean age 60 (10.6) and a MOCA score of ≥26. The podiatrists ranged in experience from 1 to 11 years. The PAID scores were collected during the initial podiatry consult and at 6 months post the consult, this scores highlight patients that may be in denial or at a level of burnout with their diabetes care needs. During the consults the podiatrists covered a range of topics relating to diabetes averaging 6 topics per consult. This education was delivered verbally in all but two cases.

**Conclusions**

This study identified that podiatrists cover a number of complex concepts during a diabetes consultation. There was a disparity between key messages both at the time of the appointment and at follow up. This has potential negative implications for self care and early identification of foot complications relating to diabetes.

### O40 Clinical utility of different offloading techniques to reduce peak plantar pressures at neuropathic forefoot ulcers sites in people with diabetes: a preliminary study

#### Georgina Frank^1^, Vanessa Nube^1^, Stefania Penkala^2^

##### ^1^NSW Health Sydney Local Health District, Sydney, NSW, Australia; ^2^Western Sydney University, Sydney, NSW, Australia

###### **Correspondence:** Georgina Frank

**Objectives**

Excessive plantar pressures on insensate feet are a major risk for developing ulcers and potential amputations. While total contact casts are the gold standard to offload they are not always practical or tolerated. This study investigated the effectiveness of different offloading interventions to reduce peak pressures at forefoot ulcer sites.

**Method**

A within-subject repeat measures design was used to investigate peak pressures between offloading interventions: Rocker post-op shoe, with a prefabricated or custom heat-moulded orthosis, and a removable total contact boot, with a prefabricated or custom heat-moulded orthosis. The Novel Pedar X in-shoe measurement system was used to collect peak pressures. Participants walked at a self- determined speed, controlled within 5% between trials. Offloading interventions were tested in a random order and normal footwear was worn on the unaffected side. Ten steps were identified for each trial and ulcer sites ‘masked’ using the Novel software and data compared between trials.

**Results**

In an evaluation of four participants, the mean reduction in peak pressure at forefoot ulcer sites was 94kPa (46%) between the post-op shoe (204 ± 25kPa) and the removable total contact boot (110 ± 31kPa). Within the post-op shoe, prefabricated and heat-moulded orthoses on average reduced peak pressures by 20% and 30% respectively. Within the total contact boot, the prefabricated and heat moulded condition on average reduced peak pressure a further 10 and 25% respectively. Preliminary results suggest individualising offloading is supported and can accommodate individual variations.

**Conclusions**

Removable total contact boots offered better forefoot offloading compared to post-op shoes. Prefabricated and heat-moulded orthoses can further reduce pressures within recommended guidelines (<200kPa or <25%). Customising offloading can provide alternate options when availability, patient compliance and functional ability may limit the use of ‘gold standard’ offloading choices.

### O41 A novel 3D printed foot model to enhance anatomical knowledge and palpation skills in podiatry students

#### Stefania Penkala, Tosin Famakinwa, Manisha Dayal

##### Western Sydney University, Sydney, NSW, Australia

###### **Correspondence:** Stefania Penkala

**Objectives**

Comprehensive anatomical knowledge is vital in podiatric education. While students can recite anatomical content translation into clinical practice is challenging. Palpation skills are often limited. This study aimed to determine if functional 3D ‘real-life’ printed foot models with graded densities of transparent and opaque materials enhanced anatomical and palpation skills.

**Method**

Three identical 3D printed foot model were developed. One model was transparent but rigid. The remaining models where opaque but more flexible with moveable metatarsophalangeal joints. First and fourth year students participated in a guided palpation session using the models and translating the information on to ‘real’ feet. Survey and focus groups data were collected. Focus group data is the focus of this paper. Focus groups were recorded and transcribed verbatim. Data was coded until unique themes where identified. The theme ‘Consistency between Models’ will be discussed.

**Results**

Six focus groups were undertaken with a total of 16 students participating. The ‘Consistency between Models’ facilitated learning. *‘The thing that I found most beneficial … the models - from transparent to opaque were all the same’* and ‘*To see how these joints all lined up and then feel it in this model …how they moved, it helped…for a better understanding of how it works*’ and *‘If you’re not feeling the process or the joint line properly at least you’ve got the clear model …you can visualize it and … transfer that image to the palpable model’*.

**Conclusions**

3D printed models can assist student confidence and anatomical knowledge and skill to palpate anatomic structures in the foot. Density and transparency variations of similar models provided additional learning by comparing and contrasting structures. Models can be used to create opportunities to explore ‘real’ skills, prior to patient involvement.

### O42 Ultrasound characteristics of the mid-portion of the Achilles tendon in symptomatic and asymptomatic runners: a systematic review

#### Prue Molyneux, Matthew Carroll

##### Auckland University of Technology, Auckland, New Zealand

###### **Correspondence:** Matthew Carroll

**Objectives**

Achilles tendinopathy is a common overuse injury in runners, yet the clinical significance and frequency of abnormal sonographic characteristics in runners remains unclear. The aim of this review was to assess literature which has employed ultrasonography to evaluate ultrasound characteristics of the mid-portion of the Achilles tendon runners.

**Method**

An electronic literature search was performed using the following electronic databases: Medline, CINAHL and SPORTDiscus. Studies published in English were included if they evaluated ultrasound characteristics associated with mid-portion Achilles tendinopathy (i.e. 2–6 cm proximal to its insertion) in runners. Methodological quality was assessed using a scale adapted from the Newcastle-Ottawa Scale.

**Results**

Six studies were identified. Three studies were case-control, two studies were cohort and one study was cross-sectional. Studies demonstrated ‘very good’ or ‘good’ methodological quality. The six included studies involved 767 running participants, of which 22 were symptomatic at the mid-portion of the Achilles tendon. The six studies reported on one or more of the following sonographic outcome measures relating to Achilles tendinopathy in symptomatic and asymptomatic runners: hyper- and hypoechogenicities, neovascularisation, Achilles tendon thickness, cross sectional area and tendon stiffness/strain.

**Conclusions**

Ultrasound characteristics are more prevalent in runners who are symptomatic, overweight and have a greater running volume. However, intratendinous changes are also evident in asymptomatic and normal weight runners, indicating the potential for adaption to increased running volume.

### O43 Student embedded clinics to improve workplace preparedness: a university and NSW Health collaboration

#### Stefania Penkala^1^, Kristy Sturday^2^, Jessica White^2^, Vanessa Nube^2^

##### ^1^Western Sydney University, Sydney, NSW, Australia; ^2^NSW Health Sydney Local Area Health Service, Sydney, NSW, Australia

###### **Correspondence:** Stefania Penkala

**Objectives**

Public Podiatry in NSW is a critically small workforce in urgent need of strategies to sustain and ‘future-proof’ the workforce. Limited workplace capacity requires innovative strategies to facilitate authentic student training opportunities and workplace preparedness and to support workplace clinical supervisors. An embedded student clinic is described and evaluated.

**Method**

An embedded ‘at risk’ patient clinic was established within a NSW Local Health District in a University and NSW Health collaboration. Concurrent patient lists were scheduled in conjoined rooms over three months. Four to five final year students attended each clinic for four-week periods and rostered through interdisciplinary clinics. A 1:5 student supervision ratio was used with students mostly working in pairs. A qualitative analysis of student experiences was undertaken via focus groups/interviews. Transcripts were transcribed verbatim and coded until unique themes where identified to evaluate their clinical experience. Patients completed a ‘satisfaction’ survey.

**Results**

Over 90% of patients completing the survey indicated satisfaction with student treatment. Twelve of fourteen students participated in interviews. Of the seven themes identified workplace preparedness and future job opportunities was central. Autonomy in the clinic was seen to support confidence and transition into practise. *‘A level of confidence to feel that I can be autonomous…opportunity to make decisions....I think reinforces the skills....and your ability to transition to practise’.* Self-reflection and gaining confidence in clinical skills in a ‘real world’ clinic and feeling part of a team were important. The ‘*team environment felt we were out in the workforce’.*

**Conclusions**

Embedded student clinics can meet patient, student and workforce needs, and sustainability. Success enabled additional clinics to be established. Careful planning is needed. Facilitating student autonomy helps promote self-regulation and workforce preparedness. A dedicated NSW Health podiatry supervisor and university academic are recommended to maintain supervision quality and sustainability.

### O44 Infection predictors for people with diabetes-related foot ulcers

#### Limin Jia^1,2^, Christina Parker^1^, Tony Parker^1^, Ewan Kinnear^3^, Patrick Derhy^4^, Ann Alvarado^1^, Flavia Huygens^1^, Peter Lazzarini^1,3^

##### ^1^Queensland University of Technology, Brisbane, Australia; ^2^Ningxia People’s Hospital, Yinchuan, China; ^3^Metro North Hospital & Health Service, Brisbane, Australia; ^4^Queensland Health, Brisbane, Australia

###### **Correspondence:** Peter Lazzarini

**Objectives**

People with infected diabetes-related foot ulcers (DFU) have a significantly increased risk of amputation; however, few studies have investigated incidence and predictors for infections in people with DFU. This study aimed to investigate the incidence and predictive factors for developing infection in patients presenting with non-infected DFU.

**Method**

This study was a secondary analysis of data collected in a large prospective state-wide diabetic foot database. Patients presenting with a non-infected DFU to one of 30 outpatient High Risk Foot Services across Queensland between 2012–2014 were included. Self-reported demographic, social determinant, medical history, foot disease history, past foot treatment, and, clinically-diagnosed DFU characteristics, type and management provided were captured at baseline. Patients were then followed up until healed or 12 months whichever came first to determine the incidence of clinically- diagnosed foot infection. Multivariate logistic regression models were used to test for predictive factors for developing infection.

**Results**

Overall, 853 patients were included; mean(SD) age 63 (13) years, 68.0% male, 90.9% type 2 diabetes and 13.6% indigenous. Foot infection developed in 40.1% of patients and there was no difference in annual incidence between DFU types (p > 0.05). Independent predictive factors for developing infection in patients with DFU were: deep ulcers (Odds Ratio 2.4); peripheral neuropathy (OR 1.8); previous foot ulcer history (OR 1.8); female gender (OR 1.5); and age in years (OR 0.98) (all p < 0.02).

**Conclusions**

Findings suggest at least 40% of people with DFU will develop an infection. Those developing an infection were more likely to have deep ulcers, peripheral neuropathy, previous DFU, be female and of younger age on presentation. Further research is recommended to investigate other potential risk factors for developing infection.

### O45 Padded socks in rheumatoid arthritis

#### Matthew Carroll, Lisa Davidtz, Angela Brenton-Rule, Mike Frecklington, Belinda Ihaka, Keith Rome

##### Auckland University of Technology, Auckland, New Zealand

###### **Correspondence:** Matthew Carroll

**Objectives**

The feet are commonly affected in rheumatoid arthritis (RA). Non-surgical interventions for RA-related foot pathology include foot health education and footwear. There is limited evidence on the role of padded socks in RA. The study aim was to compare the characteristics of four commercially available socks in people with RA.

**Method**

Thirty-five participants with RA were randomly allocated and fitted with four pairs of socks (K-Mart sport sock, Physipod® circulation sock, Drymax® Diabetic sock, Dr Comfort® crew sock). Participants walked a predetermined 100 m course at a self-selected speed. Each participant completed a 100 mm visual analogue scale relating to comfort, fit, ease of putting the sock on and off and weight. Statistically significant differences between the sock characteristics were analysed using one-way repeated measures analysis of variance.

**Results**

Participants were predominately female, with a well-established disease duration, and mean age of 67-years-old. The Drymax® Diabetic sock was significantly more uncomfortable than all socks in the study (p < 0.01). The fit of the Drymax® Diabetic sock was also significantly poorer when compared to the K-Mart sport sock and the Physipod® circulation sock (p <0.01). The Drymax® Diabetic sock was also perceived as significantly heavier than the three other sock types (p < 0.001). With respect to ease of putting socks on and off, significant differences were found between the Physipod® circulation sock, Drymax® Diabetic sock and the Dr Comfort® crew sock (p < 0.01).

**Conclusions**

The findings provide insight into the characteristics of a range of commercially available socks which may be of importance in people with RA. This information may aid clinicians in providing foot health education and footwear advice for people with RA.

### O46 Peripheral neuropathy, peripheral arterial disease, foot deformity, previous foot ulceration and previous amputation in representative inpatients

#### Peter Lazzarini^1,2^, Sheree Hurn^1^, Suzanne Kuys^2,3^, Maarten Kamp^1^, Vanessa Ng^2^, Courtney Thomas^4^, Scott Jen^5^, Jude Wills^6^, Ewan Kinnear^2^, Michael d’Emden^1,2^, Lloyd Reed^1^

##### ^1^Queensland University of Technology, Brisbane, Australia; ^2^Metro North Hospital & Health Service, Brisbane, Australia; ^3^Australian Catholic University, Brisbane, Australia; ^4^North West Hospital & Health Service, Mount Isa, Australia; ^5^West Moreton Hospital & Health Service, Ipswich, Australia; ^6^Central Queensland Hospital & Health Service, Rockhampton, Australia

###### **Correspondence:** Peter Lazzarini

**Objectives**

One in ten inpatients has foot disease; yet, no study has investigated the foot risk factors for developing foot disease in representative inpatients. This study aimed to investigate the prevalence and associates of peripheral neuropathy, peripheral arterial disease, foot deformity, previous foot ulcerations and previous amputations in representative inpatient population.

**Method**

This study was a secondary analysis of data collected as part of the Foot disease in inpatients study, a multi-site point-prevalence study of 733 representative inpatients. Self-reported socio- demographic, medical history, self-care ability and past foot treatment characteristics were obtained from all participants. The outcomes of peripheral neuropathy (PN), peripheral arterial disease (PAD), foot deformity, previous foot ulceration and previous amputation were clinically-diagnosed in all participants. Multivariate logistic regression was used to identify independent factors associated with each outcome.

**Results**

Overall, 336 participants (46.0% (95% CI: 42.4–49.7%)) had at least one outcome. PN (in 22.0% participants) was independently associated with PAD, older age, diabetes and mobility impairment (p < 0.05). PAD (21.0%) was independently associated with PN, older age, males, indigenous peoples, cancer and past surgeon treatment (p < 0.02). Foot deformity (22.4%) was independently associated with PN, older age, mobility impairment and past podiatry treatment (p < 0.01). Previous foot ulceration (9.8%) was independently associated with PN, PAD, past podiatry and past nurse treatment (p < 0.02). Previous amputation (4.1%) was independently associated with previous foot ulceration, foot deformity, cerebrovascular accident and past surgeon treatment (p < 0.01).

**Conclusions**

Nearly one in every two inpatients had a foot risk factor for foot disease. Those with foot risk factors were more likely to be of older age, male, indigenous, have diabetes, cerebrovascular accident history, mobility impairment, other foot risk factors or past foot treatment.

### O47 The habitual physical activity and sleep patterns of adults with diabetes in a regional Victorian population

#### Dimitri Diacogiorgis^1^, Byron Perrin^2^, Craig Staunton^2^, James Gerrard^2,3^, Michael Kingsley^2^

##### ^1^Ballarat Health Services, Ballarat, Victoria, Australia; ^2^La Trobe Rural Health School, La Trobe University, Bendigo, Victoria, Australia; ^3^School of Allied Health, La Trobe University, Bundoora, Victoria, Australia

###### **Correspondence:** Byron Perrin

**Objectives**

Monitoring the activity patterns in people with diabetes might help prevent foot ulceration. However, little is known about these behavioural parameters in regional Australian populations. The aim of this study was to measure the habitual physical activity and sleep patterns of regionally based people with diabetes.

**Method**

Adults with type 2 diabetes were recruited from a regional Australian population. Participants continuously wore a triaxial accelerometer (Actigraph, US) for fourteen days. The proportion of activity undertaken at low-intensity, moderate to vigorous-intensity (MVPA), daily Metabolic Equivalents (METs) and daily energy expenditure were determined by Freedson VM3 classification algorithm. The Cole-Kripke sleep algorithm was used to quantify sleep quality and duration (Actilife6; Actigraph, US). Descriptive data were summarised using means and standard deviations for continuous data and frequencies and percentages for categorical data. Independent samples t-tests were conducted to investigate differences in outcome measures according to participant history of ulceration.

**Results**

Twenty-two participants completed the study requirements, 55% male, with age 65 + 10 years, duration of diabetes 18 + 10 years and BMI of 32.8 + 7.3. Half of the participants had history of foot ulceration. Accelerometer wear time was 93 ± 6%. Energy expenditure was 9162 ± 3933 kJ/day. Proportion of daily activity in intensity bands were 81 ± 6% light and 19 ± 6% MVPA. Sleep duration and efficiency were 4.9 + 0.9 hours and 93 ± 4%, respectively. Despite not reaching statistical significance, participants with history of ulceration tended to be more active (1.53 ± 0.24METs) with greater individual variance (1.55 + 0.91METs) than those without history of ulceration (1.41 ± 0.16METs; t(20) = 1.36, p = 0.18; and 1.02 ± 0.60METs; t(20) = 1.63, p = 0.12, respectively).

**Conclusions**

Consistent with other preliminary research, despite being sedentary, people with diabetes at higher risk of ulceration maintain actual and variability in daily activity levels. These important findings will inform further research into the role activity plays in the development of ulceration, and the development of education and management plans.

### O48 Reliability of Fitbit® wearable activity monitors during walking in people with rheumatoid arthritis

#### Matthew Carroll, Lisa Davidtz

##### Auckland University of Technology, Auckland, New Zealand

###### **Correspondence:** Matthew Carroll

**Objectives**

At slower walking speeds the reliability of wearable activity monitors to measure step count has been shown to be poor. The aim of the study was to investigate the interdevice reliability of Fitbit® activity monitors to measure total step count and walking distance in people with rheumatoid arthritis (RA).

**Method**

Three Fitbit® activity monitors, mounted on the wrist, hip and knee were tested concurrently in 15 participants (13 female) with RA. Participants walked at a self-selected walking speed for two- minutes. The total number of steps taken and the total distance walked were compared between the three devices. Inter-device reliability was assessed using and intra class correlation coefficients (ICC). Statistical differences between the three devices were assessed by one-way analysis of variance.

**Results**

There was a statistically significant difference between the three Fitbit® devices for the measurement of total walking distance (p < 0.001), and no statistically significant difference between the three devices for measurement of step count (p = 0.076). When measuring total walking distance there was a fair correlation between hip vs. ankle (ICC = 0.37), a poor correlation between hip vs. wrist (ICC = 0.20), and a moderate correlation between ankle vs wrist (ICC = 0.56) measurements. When measuring step count, inter-device reliability showed a strong correlation between hip vs. ankle (ICC = 0.73), and a moderate correlation between hip vs. wrist (ICC = 0.49,) and ankle vs wrist (ICC = 0.56) measurements.

**Conclusions**

The variation between the Fitbit® monitors suggests that the anatomical location influences the reliability of the devices to measure step count and total walking distance. Clinicians who advocate the use of activity monitors in patients with RA should interpret results related to total walking distance with caution.

### O49 What types of Australians wear what types of footwear?

#### Peter Lazzarini^1,2^, Jaap van Netten^1^, Sheree Hurn^1^, Suzanne Kuys^2,3^, Maarten Kamp^1^, Lloyd Reed^1^

##### ^1^Queensland University of Technology, Brisbane, Australia; ^2^Metro North Hospital & Health Service, Brisbane, Australia; ^3^Australian Catholic University, Brisbane, Australia

###### **Correspondence:** Peter Lazzarini

**Objectives**

Guidelines recommend footwear to manage various conditions; yet, few studies have investigated the types of footwear Australians’ wear and those who wear them. The aim of this study was to investigate the prevalence and factors independently associated with different footwear types worn in a large representative Australian population.

**Method**

This study was a secondary data analysis of a cohort of 733 inpatients that were considered highly representative of a typical Australian inpatient population; 62 ± 19 years, 55.8% male, 23.5% diabetes, 4.6% indigenous. Socio-demographic, medical history, peripheral arterial disease, peripheral neuropathy, foot deformity, foot ulcer history, amputation history and past foot treatment variables were collected from all participants. Participants selected the footwear type they mainly wore outside the house in the previous year from 16 different types of footwear. Multivariate logistic regression was used to identify independent factors associated with different types of footwear.

**Results**

The main footwear worn outside were running (20.4%), thongs (14.2%), walking (13.5%), sandals (13.1%), boots (10.7%), oxford (6.9%), court (6.7%), moccasins (5.8%), slippers (2.8%), bespoke (1.7%) and no (1.7%) shoes. Independent associations with footwear included: running shoes with males; thongs with younger age, females, socio-economic status; walking shoes with arthritis; sandals with females, non-smokers; boots with males, geographical remoteness; oxford shoes with older age, males; court shoes with females, older age, non-smokers, past podiatry treatment; moccasins with females; slippers with males, chronic kidney disease; bespoke shoes with past podiatry treatment; and no shoes with younger age, peripheral neuropathy (*all p < 0.05*).

**Conclusions**

This study suggests there are common types of footwear worn outside in Australia and common types of Australians who wear those types of footwear. These findings profiling the types of people who wear different footwear may assist with targeting the implementation of guideline recommendations for footwear in future.

### O50 What factors are linked with minor and major amputations in a representative Australian population?

#### Peter Lazzarini^1,2^, Jaap van Netten^1^, Sheree Hurn^1^, Suzanne Kuys^2,3^, Maarten Kamp^1^, Lloyd Reed^1^

##### ^1^Queensland University of Technology, Brisbane, Australia; ^2^Metro North Hospital & Health Service, Brisbane, Australia; ^3^Australian Catholic University, Brisbane, Australia

###### **Correspondence:** Peter Lazzarini

**Objectives**

Minor and major amputations are reportedly performed for different clinical reasons; yet very few Australian studies have investigated the factors associated with these different amputations. The aim of this study was to investigate the independent factors associated with minor amputations and major amputations in a representative Australian inpatient population.

**Method**

This study was a secondary analysis of data collected from the *Foot disease in inpatients study*, a multi-site point-prevalence study of 733 representative Australian inpatients; age 62 ± 19 years, 55.8% male, 23.5% diabetes, 4.6% indigenous. Self-reported demographic, social determinant, medical history, self-care ability and footwear variables were obtained from all participants. Clinically- diagnosed minor and major amputation history, foot ulcer history, peripheral arterial disease (PAD), peripheral neuropathy (PN) and foot deformity were also collected from all participants. Multivariate logistic regression was used to identify independent factors associated with minor and major amputations; and were adjusted for age, sex and diabetes.

**Results**

Overall, 37 participants (5.0% (95% CI: 3.7–6.9)) had an amputation history. Minor amputation history, present in 28 participants (3.8% (2.6–5.5)), was independently associated with foot ulcer history, cerebrovascular accident history, peripheral neuropathy and foot deformity (p < 0.03). Major amputation history, present in 9 participants (1.2% (0.6–2.4)), was independently associated with foot ulcer history only (p < 0.001).

**Conclusions**

Minor amputations were linked with foot ulcers, neuropathy, foot deformity and cerebrovascular accident. Major amputations were linked with foot ulcers. These findings suggest minor amputations may be prevented by intervening in patients with neuropathy and foot deformity; whilst both amputations may be prevented by intervening in patients with foot ulcers.

### O51 New world order for Australian Podiatrists – National Disability Insurance Scheme, My Aged Care, Health Care Homes; the changing landscapes

#### Carol Mioduchowski

##### Barwon Health, Geelong, Australia

**Objectives**

Health care reform and commonwealth health policy changes are driving new funding models for all health practitioners and services. All podiatrists need to be aware how these new funding models will affect their service delivery and the profession in what has been a clearly delineated private and public health system.

**Method**

Using a narrative enquiry process, a number of case studies have been examined in the space of the National Disability Insurance Scheme (NDIS) and My Aged Care (MAC), across a number of professions in the public health sector in the Geelong area. These cases were further themed to look for key principles and learnings to assist with navigating these new systems.

**Results**

Geelong being a pilot testing site for NDIS, has provided some interesting learnings which can be applied across the health sectors. The NDIS and MAC are similar models and have provided a range of challenge’s both from an operational and professional respective. The key themes will help podiatrists and podiatry educators consider preparation for this new approach to funding health care. The themes and learning s are applicable to both public and private sectors, providing food for thought about moving into an environment of uncertainty.

**Conclusions**

The learnings of NDIS and MAC are applicable to all podiatrists. The policy context and understanding the principles will assist in business functioning in these and the new health care home model being further rolled out by the commonwealth.

### O52 Understanding where you fit in the risk profile of complaints

#### Podiatry Board of Australia

##### Podiatry Board of Australia, Melbourne, Australia

**Objectives**

Podiatrists and podiatric surgeons have been regulated nationally in Australia since the introduction of the National Registration and Accreditation Scheme (the National Scheme) on 1 July 2010. The Australian Health Practitioner Regulation Agency (AHPRA) supports each of the 14 National Boards to implement the National Scheme.

**Method**

Public safety is the primary objective of the Podiatry Board of Australia (Board). In 2014, the Board asked the AHPRA Risk-based Regulation Unit to undertake an in-depth analysis of all complaints or concerns (in the National Law called ‘notifications’) received about podiatrists and podiatric surgeons between July 2010 and June 2014.

The aim of this analysis was to gain a detailed understanding of the risk profile of complaints and concerns specifically relating to the podiatry profession.

**Results**

A total of 251 notifications were received nationally about podiatrists and podiatric surgeons (including the HPCA in NSW). The overarching themes identified through the analysis of complaints and concerns has allowed the Board to identify areas to focus regulatory activity to best protect the public. Through the presentation of these themes, it is anticipated podiatrists will gain insight into the complaints received by the Board. The key aspects of the analysis will be presented to the podiatry profession in a way that podiatrists can reflect on their individual practice.

**Conclusions**

Understanding the risk profile for the podiatry profession has allowed the Board to focus regulatory work on areas of risk for the profession. Raising awareness of arising issues will assist the profession to reflect on their own practice and to proactively reduce their risk for complaints in the future.

### O53 Minimal invasive surgery for pedal digital deformity, an audit of complications using national benchmark indicators

#### Mark Gilheany^1,2^, Dean Samaras^1,2^, Omar Baarini^1,2^

##### ^1^LaTrobe University, Melbourne, Vic, Australia; ^2^Australasian College Podiatric Surgeons, Melbourne, Vic, Australia

###### **Correspondence:** Mark Gilheany

This abstract is not included here as it has already been published.

### O54 Understanding the foot health of aged care residents

#### Georgia Coombes^1^, Alicia James^1^, Cylie Williams^1,2^

##### ^1^Peninsula Health, Mornington Peninsula, Australia; ^2^Monash University, Melbourne, Australia

###### **Correspondence:** Georgia Coombes

**Objectives**

An increasing number of older persons are admitted to residential care in Australia but little is known about the foot characteristics of this population. Foot care is commonly provided within residential facilities according to a care plan, based on the individual resident’s foot care needs.

**Method**

The study was a retrospective audit from the initial foot health screen and care plans completed by podiatrists working in residential aged care facilities at Monash Health and Peninsula Health between 2012 and 2015.

**Results**

A total of 268 resident’s foot health screen information was extracted and included within the review. There were 161 (60%) males with a mean (SD) age of 76.6 (10.0) years. At the time of audit, 12 (4%) residents had a current foot ulcer and 13 (5%) residents had previous foot ulceration. There were 159 (59%) residents with healthy skin and 124 (46%) nail pathology requiring podiatry assistance for ongoing care. Many residents had multi-morbidities which placed them at risk of foot complications including diabetes n = 67, 25%) and history of stroke (n = 52, 19%).

**Conclusions**

Understanding resident’s foot health status may assist podiatrists, foot care assistants and nursing to ensure timely, appropriate and a cost effective foot care service is provided. These audit results may be used to develop basic foot care training for care provided to residents at low risk of foot complications.

### O55 Foot orthoses for plantar heel pain: a systematic review and meta-analysis

#### Glen A Whittaker^1,2^, Shannon E Munteanu^1,2^, Hylton B Menz^1,2^, Jade M Tan^1,2^, Chantel L Rabusin^1,2^, Karl B Landorf^1,2^

##### ^1^Discipline of Podiatry, La Trobe University, Bundoora, Victoria, Australia; ^2^La Trobe Sport and Exercise Medicine Research Centre, La Trobe University, Bundoora, Victoria, Australia

###### **Correspondence:** Glen A Whittaker

**Objectives**

Plantar heel pain is a common condition affecting the foot. Foot orthoses are widely used to treat plantar heel pain, however previous meta-analyses have reported inconsistent findings. Accordingly, there is a need to conduct a systematic review and meta-analysis of the effectiveness of foot orthoses for plantar heel pain.

**Method**

Databases searched include Medline, CINAHL, SPORTDiscus, Embase and the Cochrane Library. Studies must have used a randomised design, and investigated foot orthoses for plantar heel pain. Cochrane risk-of-bias and the Grading Recommendations Assessment, Development, and Evaluation approach were used to assess the quality of studies. Outcomes evaluated were pain, function and ‘first-step’ pain. Endpoints were categorised as short (0 to 6 weeks), medium (7 to 12 weeks) or longer term (13 to 52 weeks). Mean difference or standardised mean difference and the 95% confidence intervals were calculated, and significant results were back-transformed to clinically meaningful measures.

**Results**

In the short term (0 to 6 weeks), low quality evidence found that foot orthoses do not reduce pain or improve function. In the medium term (7 to 12 weeks), moderate quality evidence found that foot orthoses were more effective than a sham at reducing pain (SMD −0.27 [−0.48 to −0.06]), however this finding did not reach the previously calculated minimal important difference value. There was no improvement in function in the medium term. In the longer term (13 to 52 weeks), very low quality evidence found that foot orthoses do not reduce pain or improve function.

**Conclusions**

There is moderate quality evidence that foot orthoses are effective at reducing pain in the medium term, however it is uncertain if the effect is clinically meaningful for patients. In the short and longer term, foot orthoses do not reduce pain or improve function.

### O56 People with patellofemoral osteoarthritis have greater foot pronation and mobility, and lower ankle dorsiflexion, compared to controls

#### Narelle Wyndow^1^, Natalie Collins^1^, Kylie Tucker^1^, Bill Vicenzino^1^, Kay Crossley^2^

##### ^1^The University of Queensland, Brisbane, Queensland, Australia; ^2^Latrobe University, Melbourne, Victoria, Australia

###### **Correspondence:** Narelle Wyndow

**Objectives**

The patellofemoral (PF) joint is commonly affected by osteoarthritis (OA). Even mild PFOA is associated with pain and functional limitations yet little is understood of its aetiological, structural and functional features. This study aimed to determine whether people with PFOA demonstrate differences in foot and ankle characteristics compared to controls.

**Method**

Foot mobility was quantified as the difference in dorsal midfoot arch height, and midfoot width, between non-weight bearing and bilateral weight bearing (50% total body weight), at 50% of the total foot length. Static foot posture was rated using the Foot Posture Index (FPI). AJTDFL range of motion was measured using the knee to wall test. FPPA was measured at the deepest part of a SLSq to 45**°**, as the angle at the knee formed by lines connecting the anterior superior iliac spine, the midpoint of the femoral condyles, and the midpoint of the malleoli. Generalized estimating equations were utilized.

**Results**

27 individuals with PFOA (18 (67%) women, mean (SD): 60 (9) yrs; 168 (8) cms; 73 (14) kgs) and 23 controls (13 (56%) women, 56 (8) years, 172 (10) cm, 72 (16) kgs) participated. The PFOA group had lower AJTDFL (p = 0.001; B = 1.88), greater arch height mobility (p = 0.002; B = 1.79), greater midfoot width mobility (p = 0.005; B = 1.71), and greater FPI (p = 0.03; B = 1.20) compared to controls. In PFOA, lower AJTDFL and greater arch height mobility were significantly associated with higher FPPA (more knee valgus) (p < 0.05).

**Conclusions**

People with PFOA have higher arch and midfoot mobility, a more pronated foot, and lower AJTDFL compared to controls. Foot and ankle features exerted differing effects on the FPPA during SLSq in PFOA compared to controls. Interventions addressing foot mobility and ankle range should be considered in people with PFOA.

### O57 Risk factors for foot ulceration in adults with end-stage renal disease on dialysis: a prospective observational cohort study

#### Michelle R Kaminski^1,3^, Anita Raspovic^1^, Lawrence P McMahon^2,3^, Katrina A Lambert^1^, Bircan Erbas^1^, Peter F Mount^4^, Peter G Kerr^2,5^, Karl B Landorf^1,6^

##### ^1^La Trobe University, Melbourne, Victoria, Australia; ^2^Monash University, Melbourne, Victoria, Australia; ^3^Eastern Health, Melbourne, Victoria, Australia; ^4^Austin Health, Melbourne, Victoria, Australia; ^5^Monash Health, Melbourne, Victoria, Australia; ^6^Melbourne Health, Melbourne, Victoria, Australia

###### **Correspondence:** Michelle R Kaminski

**Objectives**

Dialysis patients experience high rates of foot ulceration; however, the associated risk factors have been inadequately researched. This study aimed to investigate risk factors for foot ulceration in a stable dialysis cohort.

**Method**

We prospectively collected clinical, demographic, health status, and foot examination information on 450 adults with end-stage renal disease from satellite and home-therapy dialysis units in Melbourne, Australia over a 12-month period. The primary outcome was new foot ulceration. Cox proportional hazard modelling and multinomial regression were used to investigate risk factors.

**Results**

New cases of foot ulceration were identified in 81 (18%) participants. Overall, risk factors for foot ulceration were neuropathy (HR, 3.02; 95% CI, 1.48 to 6.15) and previous ulceration (HR, 2.86; CI, 1.53 to 5.34). In those without history of ulceration, nail pathology (RR, 3.85; CI, 1.08 to 13.75) and neuropathy (RR, 2.66; CI, 1.04 to 6.82) were risk factors. Whereas, in those with a history of ulceration, neuropathy (RR, 11.23; CI, 3.16 to 39.87), peripheral arterial disease (RR, 7.15; CI, 2.24 to 22.82) and cerebrovascular disease (RR, 2.08; CI, 1.04 to 4.16) were risk factors. There were 12 (2.7%) new amputations, 96 (21.3%) infections (24 osteomyelitis), 24 (5.3%) revascularisations, 42 (9.3%) foot-related hospitalisations, 30 (6.7%) transplants, and 52 (11.6%) deaths.

**Conclusions**

Peripheral neuropathy and previous foot ulceration are major risk factors for the development of foot ulceration in dialysis patients. Risk factors differ between those with and without prior ulceration. Results from this study should help reduce the incidence of foot ulceration and its associated complications in the dialysis population.

### O58 Utilisation of public podiatry and diabetes services by the Aboriginal and Torres Strait Islander community of the Central Coast of NSW

#### Matthew West^1^, Fiona Hawke^1^, Vivinne Chuter^1^, David Follent^2^

##### ^1^University of Newcastle, Callaghan, NSW, Australia; ^2^Nunyara, CCLHD, NSW, Australia

###### **Correspondence:** Matthew West

This abstract is not included here as it has already been published.

### O59 Prevalence of chronic diabetes-related foot and leg complications in the Australian Aboriginal and Torres Strait Islander Community

#### Matthew West^1^, Fiona Hawke^1^, Vivienne Chuter^1^, Shannon Munteanu^2^

##### ^1^University of Newcastle, Callaghan, NSW, Australia; ^2^La Trobe University, Melbourne, Vic, Australia

###### **Correspondence:** Matthew West

**Objectives**

Among people with diabetes, chronic lower limb complications are common, making a significant contribution to the morbidity and mortality associated with the disease. The aim of this systematic review was to determine the prevalence of diabetes-related lower limb complications in Aboriginal and Torres Strait Islander (ATSI) people in Australia.

**Method**

An electronic search of MEDLINE (from January 1966), EMBASE (from January 1980), Cochrane Central Register of Controlled Trials (CENTRAL) (The Cochrane Library 2015, Issue 2), PUBMED (from January 1966) and CINAHL (from 1982) for studies reporting the prevalence of any chronic lower limb complications in IA was conducted in August 2016. There were no language or publication restrictions. Two authors independently selected trials. One author extracted data, which was cross checked by a second author. Data are presented separately for different chronic lower limb complications.

**Results**

Eight studies published in 10 papers were included. Studies were conducted in Western Australia, Northern Territory and Queensland. Only one study was based in an urban setting. Overall, ATSI tended to experience more chronic lower limb complications at a younger age than non-ATSI. In the geographical regions studied, ATSI commonly accounted for the majority of complications despite comprising a relatively small proportion of the population. Notably, of individuals 25 to 49 years of age with diabetes, ATSI were 38 times more likely than non-ATSI to undergo major amputation and 27 times more likely than non-ATSI to undergo minor amputation.

**Conclusions**

ATSI experienced substantially more diabetes-related chronic lower limb problems at a younger age than non-ATSI and underwent markedly more amputations. There were no data available for some states and for many lower limb complications associated with significant morbidity and mortality in the general population.

### O60 What is known about the lower limb characteristics of children that display an Idiopathic Toe Walking gait pattern - systematic review

#### Antoni Caserta^1,2^, Prue Morgan^2^, Cylie Williams^2,3^

##### ^1^Monash university, Melbourne, Victoria, Australia; ^2^Monash Health, Melbourne, Victoria, Australia; ^3^Peninsula Health, Melbourne, Victoria, Australia

###### **Correspondence:** Antoni Caserta

**Objectives**

Idiopathic toe walking (ITW) is associated with ankle equinus, however no one measurement technique is used for quantification. This systematic review aims to explore how the lower limb characteristics are measures in children displaying an equinus gait associated with ITW and determine the psychometric properties of any associated measurement tools.

**Method**

Studies were collected from five databases including Ovid Medline, Medline EBESCO, Embase, CINAHL Plus, PubMed. All databases were searched from inception date until May 2016. Inclusion criteria included; participants who display equinus characteristics, up to the age of 18 years old, who are independently ambulating.

The reported psychometric properties of tools or measures used within studies were assessed for reported reliability and validity. Each study had methodology, results and reporting evaluated using the BEME criteria. This criteria allowed the recognition of limitations around reporting quality. Quality assessment was made with the Buckley quality assessment tool.

**Results**

There were 14 articles measuring equinus related ITW. Of these, only one was a randomised effectiveness trial comparing Botulinum Toxin and serial casting to serial casting only. Evidence quality was low overall, with ten studies rated as Level III, and four studies rated Level IV. Scores using the Buckley quality assessment tool ranged from 6/11 to 10/11. There were 16 measurement tools/techniques identified, only six reported reliability data and two reported validity data.

Participants commonly underwent measurement of lower limb range of motion and gait analysis however only two papers included strength as a reported measure.

**Conclusions**

Equinus is commonly associated and measured with ITW. It is unknown if there is a difference in the strength of the leg muscles contributing to or as a result of this gait style. Future research should explore the understanding of the strength profile for children with an ITW gait.

### O61 Gait and Lower Limb Observation of Paediatrics (GALLOP): development of a consensus based paediatric podiatry and physiotherapy standardised recording proforma

#### Simone Cranage^1^, Helen Banwell^2^, Cylie Williams^2,3^

##### ^1^Peninsula Health- Community Health, Melbourne, Victoria, Australia; ^2^School of Health Sciences- University of South Australia, Adelaide, South Australia, Australia; ^3^School of physiotherapy, Monash University, Melbourne, Australia, Australia

###### **Correspondence:** Simone Cranage

This abstract is not included here as it has already been published.

### O62 Oblique Talus: The Yeti of the foot world

#### Simone Cranage^1^, Naveen Nara^2^, Jennifer Powell^3^, Cylie Williams^1,4,5^

##### ^1^Peninsula Health, Community Health, Melbourne, Victoria, Australia; ^2^Royal Children’s Hospital, Melbourne, Victoria, Australia; ^3^Queensland Health, Brisbane, Queensland, Australia; ^4^School of Health Sciences, University of South Australia, Adelaide, South Australia, Australia; ^5^School of Physiotherapy, Monash University, Melbourne, Australia

###### **Correspondence:** Simone Cranage

**Objectives**

The paediatric flexible flat foot needs to be distinguished from rigid flat foot syndromes. The oblique talus is poorly understood therefore this was a systematic review aimed to determine the prevalence, diagnostic criteria and treatment of oblique talus in children.

**Method**

Two reviewers examined four databases from inception to August 2016. Search terms included child, infant, oblique, flat foot, vertical and talus (full text publications, human studies). All articles types were included. The reference lists and articles citing the included articles were also examined. The methodology, quality and risk of bias was examined and assessed.

**Results**

11465 abstracts were screened, 71 full texts reviewed, and 20 articles were included in the final extraction including one prospective study, five case reports/case series and fourteen expert opinion articles. Of the case reports/series, the largest population size was 77, however this historical article had diagnostic criteria inconsistent with that used in more recent studies including forced plantar flexion lateral radiographs. There were no reported prevalence statistics or evidence based treatment options extracted. Eleven articles reported inconsistent and/or ambiguous diagnostic criteria. There was high risk of bias on quality assessment of the included articles.

**Conclusions**

While Oblique Talus is rarely reported, it appears sporadically within the literature. Like the Yeti, there is limited evidence to conclusively prove its existence. This leaves clinicians none the wiser on evidence based treatment and more research is needed if this condition continues to be clinically observed or radiologically reported.

### O63 Medical imaging findings for plantar heel pain: it is not just the plantar fascia that is involved

#### Karl Landorf^1,2^, Shannon Munteanu^1^, Gerard Zammitt^1^, Tom Entwisle^3^, David Connell^3^, Hylton Menz^1^

##### ^1^La Trobe University, Melbourne, Victoria, Australia; ^2^Melbourne Health, Melbourne, Victoria, Australia; ^3^Imaging @ Olympic Park, Melbourne, Victoria, Australia

###### **Correspondence:** Karl Landorf

**Objectives**

Plantar heel pain (PHP) is one of the most common musculoskeletal complaints of the foot. While many patients improve with conservative management, there are some that do not, which may relate to incorrect diagnosis. The aim of this study was to investigate medical imaging findings in people with PHP.

**Method**

This study included 40 participants with unilateral PHP. The affected heel of participants was compared to the unaffected heel using plain film x-ray, ultrasound and MRI. Comparisons between heels were done using odds ratios (ORs) and 95% confidence intervals (CIs), although for brevity, only ORs from significant findings are presented below.

**Results**

On ultrasound, the following ORs were found for the affected heel: >4 for heterogeneity (i.e. >4 times more likely in the affected heel), >7 for delamination, and nearly 5 for a tear of the plantar fascia. On MRI, the following ORs were found for the affected heel: nearly 23 for hyperintensity, >12 for heterogeneity, >8 for delamination, and >6 for a tear of the plantar fascia. Additional ORs of note (that highlight other tissues that are involved) include: nearly 10 for perifascial oedema, nearly 10 for plantar fat pad oedema, and 6 for bone marrow oedema of the calcaneus.

**Conclusions**

These findings highlight that PHP does not just involve the plantar fascia. While pathology of the plantar fascia is part of the clinical picture, increased odds of bone marrow oedema and other tissue involvement was noted in this study. This may have important implications for the treatment of PHP.

### O64 The effect of foot orthoses on lower limb biomechanics in patellofemoral joint osteoarthritis

#### Jade Tan^1^, Kane Middleton^1^, Havi Hart^1^, Hylton Menz^1^, Kay Crossley^1^, Shannon Munteanu^1^, Natalie Collins^1,2^

##### ^1^La Trobe University, Melbourne, Victoria, Australia; ^2^The University of Queensland, Brisbane, Queensland, Australia

###### **Correspondence:** Jade Tan

**Objectives**

The aim of this study is to determine the immediate effects prefabricated foot orthoses versus flat insoles on lower limb biomechanics in people with patellofemoral joint osteoarthritis (PFJ OA), and to evaluate the short-term effects on pain and function to determine whether they are an acceptable intervention for PFJ OA.

**Method**

This study was a parallel group, single blinded, randomised controlled trial in a community setting for six-weeks. 26 participants with PFJ OA completed clinical testing, patient-reported outcome measures, and biomechanical testing using 3D motion capture (Vicon) during the baseline appointment. They were then followed for six-weeks to assess their pain levels and functional outcomes. The test orthosis was the commercially available prefabricated full-length Vasyli orthosis. The control device was a flat insert made of 3 mm high-density EVA.

**Results**

All participants have been recruited and all follow up data has been collected and entered into Microsoft Excel. Statistical analysis is currently being undertaken undertaken using SPSS® version 24.0 (IBM Corp, NY, USA). The primary end point was six-weeks, as this is considered to be the time of greatest effect. When the assumption of normality is met, within-subject, repeated measures (ANOVA) will be performed with significance level set at <0.05. This is to evaluate the immediate effects of prefabricated foot orthoses on lower limb biomechanics, and the short-term effects on pain and function in people with PFJ OA.

**Conclusions**

To be confirmed

### O65 Physical and mechanical therapies for lower limb pain in children with joint hypermobility syndrome: a systematic review

#### Benjamin Peterson^1^, Andrea Coda^1^, Verity Pacey^2^, Fiona Hawke^1^

##### ^1^The University of Newcastle, Ourimbah, NSW, Australia; ^2^Macquarie University, Sydney, NSW, Australia

###### **Correspondence:** Benjamin Peterson

**Objectives**

Joint hypermobility syndrome (JHS) is one of the most common heritable genetic connective tissue disorders, characterised by excessive joint range and musculoskeletal symptoms. This systematic review evaluated randomized and quasi-randomized controlled trials of non-invasive, physical and mechanical therapies in improving quality of life and reducing pain in children with JHS.

**Method**

MEDLINE, EMBASE, Cochrane Central Register of Controlled Trials, PUBMED and CINAHL were searched between March 21st and April 21st 2016 for randomised controlled trials (RCT) and quasi- RCT investigating physical and mechanical interventions for lower limb problems in children with JHS. Two authors independently screened studies for eligibility for inclusion. Three review authors independently assessed risk of bias, following criteria described in the Cochrane Handbook for Systematic Reviews of Interventions. One author extracted and analysed statistical data, which were checked by a second author.

**Results**

Two RCTs were eligible for inclusion, including a total of 86 participants, which evaluated differences between generalised versus targeted physiotherapy programs and exercising to neutral knee extension versus to the hypermobile range. Greater improvements in quality of life (QoL) were achieved by performing knee extension exercises to the full hypermobile range, measured using the Child Health Questionnaire (mean difference 9.00; 95% CI: 1.47 to 16.53). No other statistically significant differences were identified.

**Conclusions**

Children with JHS-related knee pain experienced improved QoL by performing knee extension exercises to the full hypermobile range rather than the neutral range. No benefit was found for a targeted physiotherapy program over a generalized physiotherapy program. No mechanical intervention for children with JHS has been evaluated in RCT.

### O66 Lower Limb Rehabilitation Therapies in Joint Hypermobility Syndrome - A Survey of New South Wales and Australian Capital Territory Podiatrists

#### Benjamin Peterson^1^, Fiona Hawke^1^, Lisa Newcombe^2^, Kim Hennessy^3^, Joshua Burns^4^, Davinder Singh Grewal^4^, Verity Pacey^5^, Andrea Coda^1^

##### ^1^The University of Newcastle, Ourimbah, NSW, Australia; ^2^Glasgow Caledonian University, Glasgow, G4 OBA, UK; ^3^University of Western Sydney, Campbelltown, NSW, Australia; ^4^The University of Sydney, Sydney, NSW, Australia; ^5^Macquarie University, Sydney, NSW, Australia

###### **Correspondence:** Benjamin Peterson

**Objectives**

Joint hypermobility syndrome (JHS) is one of the most common disorders of the connective tissue, characterised by excessive joint range and musculoskeletal symptoms. This survey aimed to explore New South Wales (NSW) and Australian Capital Territory (ACT) podiatrists’ experiences, knowledge and practices in relation to managing children diagnosed with JHS.

**Method**

An online survey was administered through Survey MonkeyTM with questions pertaining to clinicians’ knowledge and use of lower limb rehabilitation therapies in JHS. All members of the Australian Podiatry Association (NSW/ACT) were invited via email to participate. Descriptive statistics and correlation analyses were performed.

**Results**

Fifty-seven podiatrists completed the survey. Sixty-four percent (n = 34) reported treating JHS. During the 6 months prior to undertaking the survey, 57% (n = 19) reported treating 0–5 JHS patients and 3% (n = 1) reported treating greater than 50. Seventy-seven percent were unaware of clinical guidelines for treating JHS. Exercise therapy was implemented by 88.9% (n = 24/27), most commonly, lower limb strengthening (88.9%; n = 24/27). Orthotic therapy was used by 96.3% (n = 26/27). There were no statistically significant differences in prescription of lower limb stretching or strengthening exercises between respondents who were aware of clinical guidelines and those who were not.

**Conclusions**

Most respondents reported using interventions that have not been evaluated in randomised trials for JHS. Awareness of current clinical guidelines for JHS had no statistically significant impact on the practices of NSW and ACT podiatrists. Randomised trials and updated clinical guidelines are required to guide podiatrists’ treatment of JHS.

### O67 What makes feet so damn insensitive? A systematic review of risk factors for the development of diabetes-related peripheral neuropathy

#### Ainslie Davies^1^, Barry Matthews^1^, Jason Warnock^1^, Malindu Fernando^2^, Peter Lazzarini^3,1^, Nicola Pritchard^4^, Paul Bennett^1^, Lloyd Reed^1^

##### ^1^School of Clinical Sciences, Queensland University of Technology, Brisbane, Australia; ^2^Vascular Biology Unit, Queensland Research Center for Peripheral Vascular Disease, College of Medicine and Dentistry, Faculty of Medicine Health and Molecular Science, James Cook University, Townsville, Australia; ^3^Foot Disease Research Program, Allied Health Research Collaborative, Metro North Hospital and Health Service, Queensland Health, Brisbane, Australia; ^4^Anterior Eye Laboratory, Faculty of Healt, Institue of Health and Biomedical Innovationh, Brisbane, Australia

###### **Correspondence:** Ainslie Davies

**Objectives**

Diabetes-related peripheral neuropathy (DPN) is a well-established risk factor for diabetes-related foot ulceration. Therefore, the systematic identification of risk factors for DPN seems like an important step in developing preventative strategies for foot ulceration. The objective of this systematic review was to identify risk factors leading to diabetes-related peripheral neuropathy.

**Method**

A search was conducted of the electronic databases PubMed, Embase, CINAHL via EBSCOhost and Ovid (including MEDLINE) up to 10 November 2016. Longitudinal studies with adult participants with either type1, or type 2 diabetes were included. Outcome measures were either probable or confirmed diabetes related peripheral neuropathy using two criteria at minimum, of clinical signs or symptoms. Studies were selected by two independent reviewers.

**Results**

Of 8,709 potentially relevant studies, 40 met the inclusion criteria. Five studies were randomised controlled trials and 35 observational studies. Many of the included studies were heterogeneous due to their defined outcome of diabetes-related peripheral neuropathy. Sixteen of the included papers identified elevated blood glucose levels as a risk factor for diabetes-related peripheral neuropathy. Other risk factors identified were: duration of diabetes, height, presence of other microvascular complications, smoking and cardiovascular risk factors such as low HDL cholesterol, high triglycerides, and hypertension, biomarkers of oxidative stress, myelinated nerve fibre density and gene polymorphisms.

**Conclusions**

Multiple risk factors have been identified in this systematic review leading to the development of diabetes-related peripheral neuropathy. It is recommended that the inclusion of these risk factors in a clinical tool will assist preventative strategies for diabetes-related neuropathic foot ulceration.

### O68 Tactile direction discrimination is impaired by peripheral neuropathy

#### Sarah McIntyre^1,2^, Julia Capper^3^, Collette Coughlin^4^, Isabel Woods^4^, Frances Henshaw^5^, Richard M Vickery^2,6^, Ingvars Birznieks^2,6^, Paul P Breen^1^

##### ^1^MARCS Institute for Brain, Behaviour and Development, Western Sydney University, Sydney, NSW, Australia; ^2^Neuroscience Research Australia, Sydney, NSW, Australia; ^3^St George Hospital, Sydney, NSW, Australia; ^4^Uniting War Memorial Hospital, Sydney, NSW, Australia; ^5^School of Science and Health, Western Sydney University, Sydney, NSW, Australia; ^6^School of Medical Sciences, UNSW Australia, Sydney, NSW, Australia

###### **Correspondence:** Sarah McIntyre

**Objectives**

Peripheral neuropathy is associated with a decline in tactile sensation; typically measured as sensitivity to brief touch, or vibration applied to the skin. Task performance reflects mainly axonal loss. Neuropathy also causes increased temporal dispersion due to slowed conduction velocity. More complex tasks might enable detection of this pathological feature.

**Method**

We developed an apparent motion device for use in clinical settings, in which neighbouring skin locations are successively tapped. Four tactors 20 mm apart tapped the skin on the plantar foot or lower leg. Participants judged the direction of motion, which requires them to correctly order the sequential touch events in space and time. Data were collected from volunteers diagnosed with neuropathy (7 female, 8 male, aged 43–84) and healthy controls (8 female, 7 male, aged 53–87).

**Results**

An adaptive staircase procedure was used to determine direction discrimination performance, defined as the area under the curve (AUC), with larger values indicating slower speeds were needed to make direction judgments. On both sites tested, performance was significantly worse for the neuropathic group (foot = 130 (44), leg = 136 (39) mean (SD) AUC) than for the control group (foot = 96 (33), leg = 111 (38) mean (SD) AUC; foot: t(52) = 3.2, p = 0.002; leg: t(50) = 2.3, p = 0.024).

**Conclusions**

These results indicate that the apparent motion direction judgment task has potential diagnostic value for peripheral neuropathy. It may enable early detection of neuropathy, distinguishing of different underlying causes, and characterising disease progression.

### O69 Are Toe Pressures useful? Absolutely! A systematic review to support the clinical use of Absolute Toe Pressures

#### Katrina Richards^1^, Nicholas Taylor^1,2^

##### ^1^Eastern Health, Melbourne, VIC, Australia; ^2^La Trobe University, Melbourne, VIC, Australia

###### **Correspondence:** Katrina Richards

**Objectives**

The Absolute Toe Pressure (ATP) is a simple test used for non-invasive lower limb vascular screening. An ATP of 40 mmHg or less is thought to indicate critical limb ischaemia. At Eastern Health, a Melbourne Metropolitan health network, the Podiatry department saw an increase in requests from Vascular Surgeons to perform ATPs.

**Method**

Searches were conducted in MEDLINE, CINAHL and EMBASE from the earliest available date to August 2016. Methodological quality of included studies was assessed using the QUADAS-2 tool. Eight studies were included in the review.

**Results**

The studies varied in how they assessed low ATP and clinical outcomes. Reference standards included the ability to heal ulcers or amputation sites (1 study); clinical end points such as rest pain, ulceration, amputation or vascular surgery (6 studies); and ATP compared to angiographic findings (1 study). The studies were of low to moderate quality and were assessed as having a risk of bias in at least 1 of the 4 criteria using the QUADAS-2 tool. Most studies reported that the ATP was more accurate than other quick screening tests, although sensitivity and specificity values were mostly moderate.

**Conclusions**

The ATP may be an effective screening tool to determine a patient’s risk of a poor treatment outcome. A cut-off value of between 30 mmHg or 40 mmHg may be indicative of impaired healing and should be part of a quick screening assessment for patients with peripheral arterial disease.

### O70 Can we predict diabetes-related peripheral neuropathy without lifting a finger? Testing the Bongaerts clinical screening score tool in an Australian population

#### Ainslie Davies^1^, Derek Van Lonkhuyzen^2^, Nicola Pritchard^3^, Peter Lazzarini^4,1^, Paul Bennett^1^, Lloyd Reed^1^

##### ^1^School of Clinical Sciences, Queensland University of Technology, Brisbane, Australia; ^2^Biomedical Sciences, Faculty of Health, Institute of Health and Biomedical Innovation, Brisbane, Australia; ^3^Anterior Eye Laboratory, Faculty of Health, Institute of Health and Biomedical Innovation, Brisbane, Australia; ^4^Foot Disease Research Program, Allied Health Research Collaborative, Metro North Hospital and Health Service, Queensland Health, Brisbane, Australia

###### **Correspondence:** Ainslie Davies

**Objectives**

The Bongaerts clinical screening score (CSS) for diabetes related peripheral neuropathy (DPN) was developed in an attempt to improve screening for DPN. This study aimed to test the predictive validity of the Bongaerts models for detecting DPN in a cohort with type 1 and type 2 diabetes.

**Method**

The base model proposed by Bongaerts consisted of age, height, weight, pain or discomfort in the feet and/or legs and duration of diabetes. The clinical model consisted of the base model with additional clinical data: diastolic blood pressure, and serum creatinine levels. The two models were fitted to data from participants with type 1 diabetes (n = 145) and type 2 diabetes (n = 80) using the enter method for logistic regression. The outcome measure of confirmed DPN was diagnosed in all participants using nerve conduction studies and clinical signs and symptoms recorded as defined by the Toronto Diabetic Neuropathy Expert Group.

**Results**

Both CSS models were statistically significant, (p > 0.0001) for the outcome of confirmed diabetes related peripheral neuropathy. The base model correctly classified 74.7% and the clinical model correctly classified 73%. All of the variables in the base model were significant in this application to data. The addition of diastolic blood pressure and creatinine did not aid in model predictive validity and were not significant.

**Conclusions**

The clinical screening score proposed by Bongaerts, 2015 has predictive validity for confirmed diabetes related peripheral neuropathy. The CSS modelling does not include glycaemic control, a known risk factor for diabetes related peripheral neuropathy. Thus there is potential for improved models of clinical scoring for diabetes related peripheral neuropathy.

### O71 Defining flexible pes planus in paediatrics: a systematic review

#### Maisie Paris^1^, Helen Banwell^1^, Shylie Mackintosh^1^, Cylie Williams^2,1^

##### ^1^University of South Australia, Adelaide, South Australia, Australia; ^2^Monash University, Frankston, Victoria, Australia

###### **Correspondence:** Helen Banwell

**Objectives**

Paediatric flexible pes planus is a frequently observed foot posture, however, little consensus exists on the criteria used to diagnose this condition. This systematic review of the literature aimed to determine how paediatric flexible pes planus is defined and the psychometric properties of the measures used.

**Method**

A systematic search of electronic databases (MEDLINE [Ovid], CINAHL, EMBASE, Cochrane Library, AMED, SportDiscus, PsycINFO, Scopus and Web of Science) were conducted November 2015. Medical subject headings were exploded/combined with relevant keywords, truncated as necessary. Searches were limited to English language studies. Empirical studies were included when; the population were ≤18 years of age, the sample included individuals with reported pes planus, pes planus had been defined, and measures aimed at diagnosing pes planus had been conducted and reported. Outcomes of interest also included reported validity and reliability analysis of foot posture measures associated with pes planus.

**Results**

Of 3,273 articles screened, 19 studies met the inclusion criteria involving 13,217 participants (n range 22 to 5,866). Seventeen foot posture measures were identified, with the Chippaux-Smirak index most frequently used (n = 6). Seven measures had independent data supporting validity (Chippaux-Smirak index, radiological [talus-first metatarsal angle], Clarke’s angle, footprint angle, calcaneal inclination angle, Staheli arch index and navicular motion). Of these measures, only four had independent data supporting reliability (Chippaux-Smirak index, Clarke’s angle, footprint angle and Staheli arch index). No further measures were found to have reported reliability or validity for diagnosing pes planus in a paediatric population.

**Conclusions**

A synthesis of available literature revealed no universally accepted criteria for defining paediatric pes planus. Therefore, the ability of podiatrists to justify a pes planus diagnosis is lacking. As psychometric data was limited, continued investigation into reliability and validity of foot posture measures, particularly for the paediatric population, is warranted.

### O72 The generalisation of predicted likelihood of pain in healthy controls

#### Kerwin Talbot^1,2^, Lorimer Moseley^1,2^, Sara Jones^1,2^, Victoria Madden^1,2^, Daniel Harvie^1,2^, Valeria Bellan^1,2^

##### ^1^University of South Australia, South Australia, Australia; ^2^Body in Mind Research group, South Australia, Australia

###### **Correspondence:** Kerwin Talbot

**Objectives**

The understanding of pain has rapidly advanced over the last two decades. Pain is no longer thought of as a simplistic linear event, but rather as a complex perceptual experience composed of multiple stimuli. Despite advances in understanding pain, scientists and clinicians are still unable to successfully treat it.

**Method**

A within-subject experimental design was combined with a classical trace conditioning paradigm. Based on an a priori sample size calculation 16 healthy participants were recruited through flyers, word of mouth and the host university. The study comprised of several phases: Preparation, Calibration, Acquisition, Generalisation and Extinction. The painful stimulus (Unconditioned Stimulus, US) was painful laser stimulation. The images allocated as conditioned stimuli with the US (CS+) and conditioned stimuli without US (CS-) were hand postures at extremes of flexion and extension. The generalisation stimuli were six images of novel hand postures which varied in perceptual similarity between CS+ and CS-.

**Results**

This study supported the theory that predicted likelihood of pain will generalise to novel but similar stimuli. As the stimuli (GSs) decreased in similarity to the initial painful stimulus (CS+) so too did the predicted likelihood of pain ratings. This study clearly showed that predicted likelihood of pain generalises away from the CS+, in healthy volunteers. In particular, pain was considered more likely with CS+ than with CS- (F (1, 15) = 58.1 p = 0.00) showing a classical conditioning effect.

**Conclusions**

There is a growing body of evidence that has demonstrated the importance that association learning may have in the development and perhaps the maintenance of pain. This study has highlighted that healthy participants will use previously retained information (CS-US association) and apply it to other similar situations.

### O73 The effects of heel lifts on lower limb biomechanics: a systematic review

#### Chantel Rabusin^1,2^, Hylton Menz^1,2^, Shannon Munteanu^1,2^, Angela Evans^1,2^, Jodie McClelland^1,3^

##### ^1^La Trobe Sport and Exercise Medicine Centre (LASEM) La Trobe University, Melbourne, Australia; ^2^Discipline of Podiatry, School of Allied Health, La Trobe University, Melbourne, Australia; ^3^Discipline of Physiotherapy, School of Allied Health, La Trobe University, Melbourne, Australia

###### **Correspondence:** Chantel Rabusin

**Objectives**

Heel lifts are often used as a key therapeutic intervention for many musculoskeletal conditions. The mechanisms by which heel lifts exert their effects are poorly understood. This systematic review aims to identify, evaluate and summarise the effects of heel lifts on lower limb biomechanics.

**Method**

Peer-reviewed studies were searched using electronic databases from inception to June 2016. Studies were included if they (a) were of a cross-sectional or longitudinal study design (b) involved participants without a lower limb discrepancy or neurological condition, (c) evaluated lower limb biomechanics (temporo-spatial, kinematics, kinetics, muscle activity and plantar pressures) during walking or running, and (d) used a heel lift that was removable or an existing feature of a shoe. Methodological quality of included studies was evaluated using a modified version of the Downs and Black Quality Index. Standardised mean differences (SMDs) and 95% confidence intervals were calculated.

**Results**

Twenty observational studies investigating the effects of heel lifts on lower limb biomechanics were included. Heel lifts were found to shift centre of pressure posteriorly, decrease maximum ankle joint range of motion, decrease gastrocnemius activity, % swing phase, % single support, % double support when using a heel lift during walking or running in asymptomatic populations.

**Conclusions**

Heel lifts were found to alter a range of biomechanical parameters, however further research is required to further understand how heel lifts alter lower limb biomechanics during walking and running and to determine if these biomechanical effects translate into improved clinical outcomes.

### O74 Foot orthoses for the prevention of lower limb overuse injuries in naval recruits: a randomised trial

#### Daniel Bonanno^1,2^, Shannon Munteanu^1,2^, Karl Landorf^1,2^, George Murley^1,2^, Hylton Menz^1,2^

##### ^1^La Trobe University, Melbourne, Victoria, Australia; ^2^La Trobe Sport and Exercise Medicine Research Centre, Melbourne, Victoria, Australia

###### **Correspondence:** Daniel Bonanno

**Objectives**

Overuse lower limb injuries are common in people who participate in regular physical activity. Foot orthoses are frequently used for the prevention of such injuries but evidence for their effectiveness is limited. The aim of this study is to determine if foot orthoses reduce the incidence of lower limb injuries.

**Method**

This study was a participant and assessor blinded, parallel-group, randomised controlled trial. The trial recruited 306 participants undertaking 11 weeks of basic training at the Royal Australian Navy Recruit School, Australia. Participants were randomised to a control group (flat insole, n = 153) or an intervention group (prefabricated foot orthosis, n = 153). The primary outcome measure was the combined incidence of four lower limb injuries (medial tibial stress syndrome, patellofemoral pain, Achilles tendinopathy, and plantar fasciitis/plantar heel pain). Data were analysed using the intention to treat principle.

**Results**

Data collection is completed and all data has been entered. Data analysis will be completed in January 2017. Results will be available at this time.

**Conclusions**

This randomised controlled trial will evaluate the effectiveness of foot orthoses for the prevention of lower limb overuse injuries in naval recruits. The findings of this trial will provide best available evidence on this topic.

### O75 Duration of total contact casting in patients with acute Charcot neuroarthropathy: a retrospective cohort study

#### Danielle Griffiths^1^, Michelle Kaminski^1,2^

##### ^1^Eastern Health, Melbourne, Victoria, Australia; ^2^La Trobe University, Melbourne, Victoria, Australia

###### **Correspondence:** Danielle Griffiths

**Objectives**

Charcot neuroarthropathy (CN) is a destructive joint disease of the foot. Total contact casting (TCC) is considered ‘gold standard’ treatment for acute CN. There is limited evidence on the duration of TCC for resolution of CN in Australia. Our studies goal was to evaluate the average duration of TCC.

**Method**

A retrospective analysis over three years (4 January 2012 to 4 January 2015) has been commenced on all patients with acute Charcot neuroarthropathy enrolled in the High Risk Foot Clinic (HRFC) at Maroondah Hospital. Data collection has involved reviewing hospital medical records from the following sources: medical imaging, pathology, and all tabs in the Clinical Patient Folder.

**Results**

Initial results of the data will be presented.

Data will inform us of the average duration of TCC in patients with acute CN.

The secondary body of results will investigate relationships between duration of TCC treatment and (i) participant characteristics, (ii) location of Charcot foot, (iii) skin temperature, (iv) ambulation status, (v) stage of CN when beginning immobilisation, and (vi) adjunctive treatment while in TCC.

Conference attendees will be interested to observe a local population and benchmark.

**Conclusions**

Determine the average time for TCC treatment of acute CN.

Identify if TCC duration is affected by patient characteristics, location of CN, skin temperature, ambulation, stage of CN, and adjunctive treatments.

Identification of TCC complications and causes, and critically assessing whether these complications are avoidable or can be reduced.

